# T cell-mediated SIV dissemination into the CNS: a single-cell transcriptomic analysis

**DOI:** 10.21203/rs.3.rs-6537361/v1

**Published:** 2025-05-09

**Authors:** Xiaoke Xu, Meng Niu, Benjamin G. Lamberty, Katy Emanuel, L. Daniel Estrella, Howard S. Fox

**Affiliations:** University of Nebraska Medical Center; University of Nebraska Medical Center; University of Nebraska Medical Center; University of Nebraska Medical Center; University of Nebraska Medical Center; University of Nebraska Medical Center

**Keywords:** CD4+ T cells, Acute SIV infection, scRNA-seq, HAND

## Abstract

**Background:**

CNS infection by HIV-1 contributes to neuroinflammation, cognitive impairments, and the establishment of viral reservoirs. Although HIV-1 is known to enter the brain early in infection via “Trojan horse” leukocytes, including infected monocytes and CD4+T cells, the specific cellular phenotypes facilitating this process during acute infection remain incompletely characterized.

**Objective:**

This study aims to characterize the roles of brain lymphocytes during acute infection and primary CD4+ T cell phenotypes seeding the SIV to the CNS.

**Methods:**

scRNA-seq was performed on brain and blood cells of three acutely SIV-infected rhesus macaques. The transcriptomic data were analyzed using bioinformatics approaches and validated through in vitro co-culture assays and re-analysis of a publicly available scRNA-seq dataset.

**Results:**

We found a distinct cell cluster in the brain co-expressing myeloid and lymphoid genes, suggesting brain myeloid cells may engulf CD4+ T cells entering from the blood. This finding was confirmed in by coculture studies of macrophages and T cells, and identified specific chemokines could distinguish such cells. Acute infection induced an increase in proliferating CD4+ cytotoxic-like T cells, which had high expression of viral entry receptors and adhesion molecules, indicating their role in CNS infection. Additionally, transcriptomic analysis revealed upregulation of cytotoxic genes, MHC class II molecules, ISG15, and USP18 in brain lymphocytes, indicating a robust immune response to acute infection.

**Conclusion:**

Our findings suggest that CD4+cytotoxic-like T cells represent a key lymphocyte subset responsible for initiating SIV entry into the brain and triggering neuroinflammatory processes. Furthermore, interactions between infiltrating lymphocytes and brain-resident myeloid cells may facilitate viral propagation within the CNS.

## Introduction

Human Immunodeficiency Virus (HIV) is an enveloped retrovirus that harbors two copies of a single-stranded RNA genome. It leads to acquired immunodeficiency syndrome (AIDS) by severely compromising the immune system. HIV continues to pose a significant global health challenge, affecting individuals, communities, and societies at large. As of 2022, an estimated 39 million people worldwide are living with HIV (PWH) (1). As of the latest epidemiological study in 2021, there are approximately 1.2 million PWH in the United States (2). HIV invades the brain during the acute phase of the systemic infection and can establish a latent reservoir pool as well as lead to neurocognitive dysfunction, even in the presence of antiretroviral therapy (ART). Unveiling the cellular and molecular mechanisms for infection of the brain, establishment of a reservoir, and damaging the brain that might lead to preventative or therapeutic methods is essential. In this effort, we employed a Simian Immunodeficiency Virus (SIV) infected rhesus macaque model, which can mimic HIV infection of people to understand the lymphocyte involvement in this process (3–5).

Using the SIV/rhesus macaque model, the progression of viral infection after mucosal (vaginal and rectal) exposure has been studied in detail (6, 7). CD4 + T cells are recruited to the initial site of infection, which spreads locally within the tissue. Dissemination of infection then occurs via the lymphatic system (8). Similar effects, without the initial localization of infection, occur after intravenous viral inoculation (9). Pronounced infection can then be found in the gut lymphoid tissue, which then suffers a rapid depletion of CD4 + T cells, found in both this model and in PWH (9, 10). In the absence of treatment, systemic depletion of CD4 + T cells occurs leading to immune deficiency and the development of AIDS. Once this occurs, despite the administration of ART, full restoration of CD4 + T-cells is not achieved in PWH (11). Multiple mechanisms contribute to the depletion of CD4 + T-cells (12), including killing infected cells by HIV/SIV-specific CD8 + T-cells (13), cytolysis triggered by viral release (9), and programmed cell death (14). The cytotoxic virus proteins and the inflammatory environment could also trigger uninfected cells’ death (15, 16).

Infection of the brain is less clear-cut. Brain infection is thought to be initiated by the “Trojan horse” mechanism, by which infected immune cells from the blood seed the CNS infection (17, 18). The primary targets for HIV- and SIV-infection in the CNS are characterized as cells of the myeloid lineage. In acute brain infection in SIV-inoculated monkeys, the infected brain cells are predominately perivascular macrophages (19). Both macrophages and microglia can be found to harbor HIV and SIV as well as, if encephalitis (SIVE or HIVE) develops, myeloid-derived multinucleated giant cells (19–24). Given the recognition that myeloid cells are the predominant infected cells in the brain in HIV and SIV infection studies on HIV infection of the brain have focused on these cells. However, in the blood the dominant SIV/HIV infected cell type is the CD4 + T cell. Thus, the true nature of the infected cell type responsible for brain invasion is not known. Studies have found that blocking a monocyte and lymphocyte adhesion molecule could reduce the invasion of HIV to the gut and central nervous system (CNS) (25). It has been found that lymphocyte-mediated central nervous system inflammation was concomitant with HIV/SIV infection, thus serving as an early source of neuroinflammation (26).

The two broadly classified T-cell lymphocyte phenotypes, CD4 + and CD8 + T-cells are present in the uninfected and HIV/SIV-infected brains at varied amounts. CD4 + T cells, as the main target for infection in the blood and the lymphoid system, may contribute to neuroinvasion of HIV/SIV, whereas CD8 + T cells, known to mediate antiviral functions, are more likely to promote neuroinflammation and neuropathogenesis (27, 28). However, this broad classification might oversimplify the brain lymphocyte phenotypes and their roles in HIV/SIV infection. Recent studies found that a unique CD4^dim^CD8^bright^ lymphocyte phenotype could mediate neuroinvasion and anti-HIV responses (29, 30). Those findings highlight the potential contributions of the various brain lymphocyte phenotypes in the HIV/SIV-infected brains.

Therefore, this study used high-throughput single-cell RNA sequencing (scRNA-seq) technique to identify and characterize varied lymphocyte phenotypes early after viral inoculation in SIV-infected as well as uninfected rhesus macaque brains. In the characterization of brain lymphocytes, we found an interesting cluster with co-expression of myeloid and lymphoid marker genes on scRNA-seq. Given that the phagocytosis/efferocytosis/cell fusion between macrophages and infected T cells might serve as one of the SIV/HIV cellular transmission mechanisms, we further validated and characterized this observation in a macrophage-T cell (Mac-T) cocultivation system. In addition, we also performed scRNA-seq of CD4 + lymphocytes in the blood at the same point to examine the relationship of the dominant viral targets in blood to what is found in the brain. We found that the CD4 + cytotoxic-like T cells identified in both blood and brain might be the primary T-cell phenotype seeding the SIV to the brain, which was further confirmed by integrating the dataset from another brain lymphocyte study with a longer time of acute SIV infection (21 days) (31).

## Materials and methods

### Experimental Design

To investigate the phenotypes of primary T cells that may facilitate SIV entry into the brain, as well as the responses of brain-resident lymphocytes during acute infection, we utilized a nonhuman primate model. Three independent biological replicates of adult rhesus macaques (Macaca mulatta) were intravenously inoculated with SIVmac251 and sacrificed at 12 days p.i. to capture early viral dynamics. Three uninfected macaques served as negative controls. Animals were randomly assigned to the infected or control groups. Immune cells were isolated from the six brain tissue samples (the three SIV-infected and three uninfected animals) and CD4 + T cells were isolated at the same time point from peripheral blood samples (from the three infected and one of the uninfected macaques). Single-cell RNA sequencing (scRNA-seq) was performed using the 10× Genomics Chromium platform for both compartments. In order to examine a later timepoint p.i., a published dataset (31) comprising four SIV-infected rhesus macaques and two uninfected animals was downloaded and used for comparative analyses.

To assess the biological validity of a distinct Myeloid-T cell population observed in scRNA-seq data, we conducted an in vitro co-culture experiment using human Jurkat T cells and human monocyte-derived macrophages (MDMs). Duplicate samples were prepared for each of the following three conditions: (1) co-culture with Jurkat cells pretreated with Camptothecin (to induce apoptosis), (2) co-culture with Jurkat cells pretreated with anti-CD95 antibody (Fas-mediated apoptosis), and (3) co-culture with untreated Jurkat cells. As a control for potential doublet artifacts during single-cell encapsulation, an additional fourth condition involved the physical mixing of Jurkat cells and MDMs immediately prior to loading on the 10× Genomics platform without prior co-culture. All eight samples from these four experimental conditions were processed for scRNA-seq using the 10× Genomics Chromium platform.

#### Animals

The six male adult rhesus macaques used in this study were purchased from PrimGen (Hines, IL) and New Iberia (LA) and tested negative for the indicated viral pathogens: SIV, SRV, STLV1, Herpes B-virus, and measles; and bacterial pathogens: salmonella, shigella, campylobacter, yersinia, and vibrio. Macaques were housed in compliance with the Animal Welfare Act and the Guide for the Care and Use of Laboratory Animals in the NHP facilities of the Department of Comparative Medicine, University of Nebraska Medical Center (UNMC). The primate facility at UNMC has been accredited by the American Association for Accreditation of Laboratory Animal Care International. The UNMC Institutional Animal Care and Use Committee (IACUC) reviewed and approved of this study under protocols 19–145-12-FC and 16–073-07-FC. Animals were maintained in a temperature-controlled (23 ± 2° C) indoor climate with a 12-h light/dark cycle. They were fed Teklad Global 25% protein primate diet (Envigo, Madison, WI) supplemented with fresh fruit, vegetables, and water and libitum. The animal care and veterinary personnel observed the monkeys twice daily to check their health status. Three of the six animals were intravenously inoculated with a stock of SIVmac251 to establish acute SIV infection (93T, 94T, and 95T). The other three macaques were uninfected (92T, 104T, and 111T) and used as control. Virus stocks were provided by the Virus Characterization, Isolation, and Production Core at Tulane National Primate Research Center. Our previously published paper has reported the viral loads of plasma and different brain regions in the three infected animals (i.e. 93T, 94T, and 95T) that were also used in the study (32).

#### Isolation of immune cells from the brain

Twelve days after viral inoculation, a necropsy was performed on deeply anesthetized (ketamine plus xylazine) animals, following intracardial perfusion with sterile PBS containing 1 U/ml heparin. Brains were harvested, and approximately half of the brain was taken for cell isolation. Immune cell-enriched cellular isolation from the brain was performed using our previously described procedure (33). Briefly, the brain was minced and homogenized in cold Hank’s Balanced Salt Solution (HBSS, Invitrogen, Carlsbad, CA, USA). After being centrifuged, the brain tissue was digested at 37° C in HBSS containing 28 U/ml DNase I and 8 U/ml papain for 30 minutes. After digestion, the enzymes were inactivated by adding 2.5% FBS, and the cells were centrifuged and resuspended in cold HBSS. The cell suspension was brought to a final concentration of 20% Percoll by the addition of 90% Percoll (GE HealthCare, Pittsburg, PA, USA) and centrifuged at 4° C for 15 minutes at 550 × g. The cell pallet at the bottom was resuspended in HBSS and passed through a 40 μm strainer to remove cell clumps and/or aggregates. Cells were again pelleted by centrifugation. A final wash was performed before the resulting cells were quantified on a hemocytometer and Coulter Counter Z1.

The brain cells were cryopreserved before the cell sorting and scRNA-seq. Specifically, after isolation as above, the cells were centrifuged at 4° C at 550 × g for 5 minutes, and the supernatant was removed. The pellet was dissociated by tapping and then resuspended by the dropwise addition of a solution of 4° C 10% DMSO in FBS at a concentration of 10^6^ cells per milliliter. Cells were transferred to cryopreservation tubes and slowly controlled freezing at −80° C. After 24 hours, cryotubes were transferred to liquid nitrogen for long-term storage.

#### scRNA-seq sample preparations for studying brain immune cells in acute SIV infection

In our prior analysis of brain immune cells, we isolated cells using positive selection for CD11b and/or CD45 selection (32). In order to capture more lymphocytes for analysis, we repeated the isolation of cells for scRNA-seq analysis using just CD45 selection. Samples of cryopreserved brain cell isolates, stored in liquid nitrogen described above, were rapidly thawed in a 37° C water bath. The cell recovery procedures were well described in our previous publications (33). After the recovery, cells were washed and counted by Coulter Counter Z1. Once cell concentration was known, cells were transferred to ice-cold PBS, staining with Live/Dead and PE-labeled anti-CD45 antibody (Biolegend, San Diego, CA). The live cells that were CD45 + were collected for scRNA-seq capture and library preparation.

#### Isolation of CD4 + T cells from the blood

Freshly isolated EDTA-anticoagulated blood was centrifuged, and the cells washed with PBS and then stained with UV-blue live/dead for 30 minutes at 4° C. After being blocked with FACS buffer for 3 minutes, the cells were centrifuged and resuspended in the antibody cocktail mixed in Brilliant Stain buffer (Catalog# 563794, BD Biosciences, San Jose, CA, USA) for 45 minutes. After incubation, red blood cell (RBC) lysing buffer was added to the sample and the cells were pelleted at 300 × g for 8 minutes. After centrifugation, the cells were resuspended in RBC lysing buffer, which was repeated to completely remove RBCs. The cells were then pelleted and resuspended in MACS buffer for FACS sorting. The antibody cocktail included V500-labeled anti-CD3 antibody (Catalog# 561417, BD Biosciences, San Jose, CA, USA), BV786-labeled anti-CD4 antibody (Catalog# 563881, BD Biosciences, San Jose, CA, USA), PE-CF594-labeled anti-CD20 antibody (Catalog# 562295, BD Biosciences, San Jose, CA, USA) The selection of cells was based on the size, singlet, live, and expression of CD20, CD3, and CD4. The CD20-positive cells were excluded, and the CD20-negative cells that were positive for CD3 and CD4 were collected for scRNA-seq library preparations.

### Jurkat cell culture and treatments

A human T-cell cell line (BCL2 Jurkat, ATCC CRL-2899) was obtained from ATCC and cultured in RPMI 1640 medium (Catalog# SH30027.02, Hyclone, Logan, UT, USA) supplemented with 10% fetal bovine serum (Catalog# F8067, Sigma-Aldrich, St. Louis, MO, USA) and 1% antibiotic-antimycotic (Catalog# SV30079.01, Hyclone, Logan, UT, USA). The Jurkat cells were seeded in 24-well plates at a density of 300,000 cells per well one day before the experiment. On the day of the experiment, apoptosis was induced in designated groups using either 1 μg CD95 (Catalog# 16–0958-81, Invitrogen, Carlsbad, CA, USA) or 10 μM camptothecin (Catalog# J62523.MD, Thermo Scientific, Ward Hill, MA) for 3 or 6 hours. Following incubation, apoptosis was assessed using the Alexa Fluor 488 Annexin V/Dead Cell Kit (Catalog# V13241, Invitrogen, Carlsbad, CA, USA) according to the manufacturer’s instructions. Prepared samples were then analyzed using an LSR II flow cytometer (BD Biosciences, San Jose, CA, USA).

#### Monocyte-derived macrophages (MDM) and T cells cocultivation

Monocytes were obtained from the University of Nebraska Medical Center Elutriation Core Facility under IRB approval and isolated from the peripheral blood mononuclear cells (PBMCs) of healthy donors. They were cultured in 6-well plates (3 million cells per well) or 12-well plates (1 million cells per well) using pre-prepared MDM culture medium for one week before the experiment. The MDM culture medium consisted of 50 mL macrophage serum-free medium (Catalog# 12065–074, Gibco, Grand Island, NY, USA), 500 μL HEPES (Catalog# 15630–080, Gibco, Grand Island, NY, USA), 50 μL Gentamicin (Catalog# G1397, Sigma-Aldrich, St. Louis, MO, USA), 5 μL Ciprofloxacin (20 mg/mL dissolved in 1% HCL, Catalog# 16626133, Honeywell Fluka, Charlotte, NC, USA), 500 μL Nutridoma (Catalog# 21952600, Roche, Indianapolis, IN), and 5 μL M-CSF (Catalog# 300–25-250UG, Gibco, Grand Island, NY, USA), which was filtered using Millipore Steriflip filter (Catalog# SCGP00525, Sigma-Aldrich, St. Louis, MO, USA) before use. Jurkat cells were plated one day prior to the experiment. On the day of the experiment, apoptosis was induced in designated Jurkat cell groups using either 1 μg anti-CD95 or 10 μM camptothecin for 3 hours. After centrifugation and removal of the supernatant containing CD95 or camptothecin, apoptotic or non-apoptotic Jurkat cells were cocultured with MDMs at a 10:1 ratio in MDM culture medium for 1 hour. The supernatant was then removed, and the cells were washed three times with pre-warmed PBS. For scRNA-seq and flow cytometry, the cells were dissociated from the plate by incubating with TrypLE (Catalog# 12605–010, Gibco, Grand Island, NY, USA) at 37°C for 10 minutes, followed by scraping. Cells designated for scRNA-seq were resuspended in PBS (Catalog# 14190–136, Gibco, Grand Island, NY, USA) containing 0.04% BSA (Catalog# 130–091-376, Miltenyi Biotec, Bergisch Gladbach, Germany), while those for flow cytometry were resuspended in FACS buffer (Catalog# 00–4222-26, Invitrogen, Carlsbad, CA, USA). For microscopy, MDMs were cultured on 12-well plates with glass coverslips (Catalog# 12541006, Fisher Scientific, Pittsburg, PA) and fixed with 4% paraformaldehyde (PFA) at room temperature for 15 minutes after cocultivation. To facilitate visualization in flow cytometry and microscopy, Jurkat cells and MDMs were stained with CellTracker Deep Red (Catalog# C34565, Invitrogen, Carlsbad, CA, USA) and CellTracker Green (Catalog# C7025, Invitrogen, Carlsbad, CA, USA), respectively, for 45 minutes before apoptosis induction and cocultivation. After staining, the cells were washed with prewarmed PBS three times. For microscopy, the cells were also stained with DAPI (Catolog# D1306, Invitrogen, CarIsbad, CA, USA) for 5 minutes before mounting by ProLong^™^ Gold antifade reagent (Catalog# P36930, Invitrogen, Eugene, OR, USA). Flow cytometry was performed using an LSR II flow cytometer (BD Biosciences, San Jose, CA, USA), while the colocalization of MDMs and T cells was captured at 25x water-immersed Olympus FVMPE-RS multiphoton laser scanning microscope (Evident Corporation, Waltham, MA, USA). Z-stacked images were obtained at 1024×1024-pixel size with excitation/emission wavelength of 647/665 nm (CellTracker Deep Red), 498/517 nm (CellTracker Green), and 359/461 nm (DAPI). The number of steps for z-acquisition was determined for each cell independently but each step was set to 0.5 μm. The z-stacked images were further processed with Fiji ImageJ for orthogonal view and adding the scale bar.

#### scRNA-seq library preparation

Post-sorting, isolates were concentrated to approximately 1000 cells per μL and assessed by trypan blue for viability and concentration. Brain immune cells and blood CD4 + T cells are isolated from the acutely infected macaques that were processed using the 10× Genomics (Pleasanton, CA, USA) Chromium 3’ v3 GEM kit targeting 8000 cells. And cocultivation study used 10× Genomics GEM-X Universal 3’ Gene Expression v4 4-plex kit targeting 5000 cells. Based on the targeting cell number, the ideal volume of cells was loaded onto the 10× Genomics Chromium GEM Chip and placed into the Chromium Controller for cell capturing and library preparation. In short, this occurs through microfluidics and combining with Single Cell 3’ Gel Beads containing unique barcoded primers with a unique molecular identifier (UMI), followed by lysis of cells and barcoded reverse transcription of RNA, amplification of barcoded cDNA, fragmentation of cDNA to 200 bp, 5’ adapter attachment, and sample indexing as the manufacturer instructed with version 3 reagent kits. The prepared libraries were sequenced using Illumina (San Diego, CA, USA) Nextseq550 and Novaseq6000 sequencers. The sequences have been deposited in NCBI GEO (accession number: GSE293543).

### Bioinformatics

#### Preprocessing with 10× Genomics pipelines

The sequenced brain and blood samples were processed using the 10× Genomics Cell Ranger pipelines (7.1.0). Specifically, the scRNA data were demultiplexed and aligned to the customized Mmul10 rhesus macaque reference genome (NCBI RefSeq assembly) and a chromosome representing the SIV genome, as described.(31) The scRNA-seq data from the MDM-T cell cocultivation experiments were processed using the 10× Genomics Cell Ranger pipelines (9.0.1) with GRCh38 human reference genome for alignment. Given the on-chip multiplexing was applied in library preparation, we used cellranger multi for processing the fastq files and generating count matrix. The counting summary statistics generated by 10× Genomics for aforementioned scRNA-seq data are shown in **Suppl. Table. 1**. The downstream analyses were implemented with R (version 4.3) and Python (version 3.12.7). The code used for analyzing the data in this study was deposited in this GitHub repository: https://github.com/Howard-Fox-Lab/Brain_lymphocytes_acute_SIV_infection.

#### Identification and characterization of brain immune cells and blood CD4 + T cells

We firstly combined our previous published data (32) from brains (GSE253835) with those newly sequenced brain data using Seurat R package (version 4.4.0) (34) to generate a single Seurat object. The four samples from the blood were merged to generate a separate Seurat object. Then, quality controls were performed for UMI counts, gene counts, and mitochondria percentage. We kept the cells from the blood and brain with UMI counts from 400–20000, gene counts from 400–10000, and mitochondria read percentage less than 15%. This filtering step removed ~ 9.7% of cells from the brain and ~ 3.1% of cells from the blood.

After removing the low-quality cells, we normalized the data. The normalization method used for the brain and blood dataset was normalized by natural logarithm of (x + 1), implemented by the NormalizeData function in Seurat. After normalization, we performed several functions in Seurat to reduce dimensionality, including FindaVariableFeatures, ScaleData, and RunPCA. Curated rhesus macaque genes (**Suppl. Table. 2**) filtered the features used for PCA. Then, we used the Seurat implementation of Harmony (35) to remove the batch effect between samples processed in different batches. This was followed by running the FindNeighbor, FindClusters, and RunUMAP functions, which used the first 20 PCs (determined by the ElbowPlot) and 0.2 as resolution. The other parameters that were not mentioned were set as default. To characterize each cell cluster, we detected differentially expressed genes (DEGs) by using the FindAllMarkers function (parameters: only. pos = TRUE, min. pct = 0.25, logfc. threshold = 0.25, test. use = “Wilcox”). The clustering based on all sorted brain cells generated 12 clusters, most of which were myeloid cells. Among those 12 clusters, one cluster had a myeloid and lymphoid cell marker coexpression, indicating those cells might be doublet. Therefore, we implemented the DoubletFinder R package(version 2.0.4)(36) to infer the doublet on a sample basis. We first used no ground-truth method to find the optimal pK value through the paramSweep, summarizeSweep, and find.pK functions. For the paramSweep function, we used the first 20 PCs and set ‘sct’ as false. The optimal pK with the largest value of mean-variance normalized bimodality coefficient (BCmvn) was selected. Then, we estimated the homotypic doublet proportion (nExp value), which depends on cell loading densities into the 10X genomics device and the cell type annotations. We used our initial clustering information as cell type annotations. Given our 10X single-cell experiments targeting 8000 cells and the recovery cell number was ~ 5000 for each sample, the estimated percentage of doublet was set to 0.039 (3.9%) for this estimation. Finally, doublet inferring was implemented using the doubletFinder function (parameters: PCs = 1:20, pN = 0.25, pK = optimal pK found in each sample, nExp = nExp value estimated for each sample, reuse.pANN = False, sct = False).

The clustering for sorted blood cells generated 11 clusters, most of which were lymphoid cells. Given the scope of this study, we kept the cell clusters with the expression of lymphocyte markers (e.g. CD3D, CD3G, CD3E, IL7R, TCF7) and removed other cell clusters from the downstream analyses for both brain and blood datasets. In total, we identified 26,340 brain lymphocytes and 25,808 blood lymphocytes. To cluster lymphocytes into distinct cell subsets, we followed the general procedures outlined by Smillie et al.,(37) which included subsetting lymphocyte clusters and performing the FindaVariableFeatures, ScaleData, RunPCA, RunHarmony, FindNeighbors, FindClusters, and RunUMAP functions for the subset lymphocyte clusters in brain and blood. The parameters set for rerunning those functions were the same as above—the subsetting and reclustering generated eight lymphocyte clusters in the brain and blood, respectively.

We then performed DEG analyses for newly identified lymphocyte clusters in blood and brain by using the Wilcoxon rank sum test. The statistical thresholds were set in the same way as mentioned. We also screened the expression of markers for different lymphocyte phenotypes by using FeaturePlot and VlnPlot functions in the Seurat package. To characterize the lymphocyte by CD4 and CD8 expression, we calculated the CD4 to CD8B (more specific marker for CD8 + cells) ratio. This ratio was calculated by using the formula shown below:

$$\text{CD}4\;\text{to}\;\text{CD}8B\;\text{ratio}=\frac{\text{log}2(\text{normalized}\;\text{CD}4\;\text{counts}+1)}{\text{log}2(\text{normalized}\;\text{CD}8B\;\text{counts}+1)}$$

The CD4 to CD8B ratio was calculated for each of the cells, embedded in the metadata of the Seurat objects, and plotted through the FeaturePlot function for visualization.

### Annotation validation with automatic annotation tool

In addition to the manual annotation for each brain and blood lymphoid cell cluster by known markers, we also used one of the scRNA-seq automatic annotation tools, Single-cell Mayo Map (scMayoMap) (38). The DEGs of each brain or blood cluster detected by the FindAllMarkers function in the Seurat were used as input for the scMayoMap function. The tissue parameter for this function was set as blood for both brain and blood samples given that the lymphocytes in the brain are originally from the peripheral blood. The annotation results were visualized by the scMayoMap.plot function.

### Study of SIV-positive cells in brain and blood

The SIV-positive cells were found in infected animals’ blood and brain immune cells. To understand the primary infected lymphocytes seeding SIV into the CNS, we subsetted all the brain and blood lymphocytes found with SIV mRNA transcripts for clustering. The workflow of using the subset dataset for reclustering was the same as mentioned above. However, for SIV + cells, we used the first 10 PCs (determined by the ElbowPlot) and 0.1 as the resolution in FindNeighbors, FindClusters, and RunUMAP functions.

### Study of brain and blood lymphocyte connections

We merged the brain and blood lymphocytes to understand the connection between the brain and blood dataset. We reperformed the FindaVariableFeatures, ScaleData, RunPCA, RunHarmony, FindNeighbors, FindClusters, and RunUMAP functions on the merged dataset. We found two phenotypes (CD4 + CTLs and TRM) in the brain showing a strong connection with another two phenotypes (CD4 + CTLs and CD4 + EM) in the blood by visualizing the new and old clustering information in Sankey diagram using networkD3 package (version: 3.0.4). To understand the differences between brain and blood for those cells, we performed DEG analysis for CD4 + CTLs and CD4 + EM (or CD4 + TRMs) between blood and brain using the FindMarkers function in seurat. Then, the genes with log_2_FC > 0.1 were used for GSEA, implemented by clusterProfiler package (version: 4.10.1) (39). The gseGO function was used for enriching genes to gene ontology-biological process (GO-BP) database, and the other parameters were set as: OrgDb = org.Mmu.eg.db, minGSSize = 100, maxGSSize = 500, pvalueCutoff = 0.05, eps = 0. The ggplot2 (version: 2.3.5) was used for the visualization of GSEA results. We then performed trajectory and pseudotime analyses on integrated datasets through the monocle3 R package (version 1.3.7)(40–42). The trajectory graph was inferred by the learn_graph function and fitted to the UMAP generated by Seurat. The visualization of UMAP embedding with differentiation path was through the plot_cells function. The label_cell_group, label_branch_points, and label_roots were disabled for plotting. Then, the CD4 + Naïve T cell cluster was set as the root node for ordering other lymphocytes in their pseudotime, which was visualized in the boxplot by ggplot2.

### Comparison of 12-days and 21-days acute SIV infections

Four infected (GSM6900144, GSM6900146, GSM6900148, GSM6900150) and two uninfected samples (GSM6900141 and GSM6900143) from a publicly available dataset (GSE221815)(31) were aligned to our customized reference genome by 10× Genomics pipeline. Then, they were merged with our three infected and uninfected brain samples, and the aforementioned workflow in Seurat was followed to generate clusters. The markers between clusters were found through the FindAllMakers function in Seurat, which was used for characterization. To understand the upregulated and downregulated genes for brain lymphocytes in 21 days of SIV infection compared to 12 days, we generated pseudobulk for individual samples using the AggregateExpression function in the Seurat. Since Myeloid-T cells could barely be found in the 21-day dataset, they were excluded from this DEG analysis. Then, we performed DEG analysis on the pseudobulk data using DESeq2 as a statistical test method. To study the SIV + cells, cells with SIV transcript detected from 12 days of infected brain and blood samples and 21 days of infected brain samples were identified and merged for clustering and analysis. The SIV + cells from the Myeloid-T cell cluster were excluded before clustering and other analyses.

### Analysis of scRNA-seq data from MDM-T cell cocultivation system

All eight samples from four different groups were integrated into a single Seurat object. Low-quality cells were filtered out based on the following criteria: UMI count < 1000, gene count < 500, and mitochondrial content > 15%. After filtering, 42,390 cells remained for downstream analysis. We followed a standard scRNA-seq analysis workflow in Seurat, including normalization using the LogNormalize method, identification of highly variable genes, data scaling, and PCA using the top 2,000 most variable genes. The UMAP was performed using the first 30 PCs, and clustering was conducted with a resolution of 0.2. To correct for batch effects, we applied Harmony before running UMAP and clustering. Marker genes for each cluster were identified using the FindAllMarkers function with the same parameter settings. For further analysis, the processed Seurat object was converted to an h5ad file for visualization in the Scanpy Python package (version 1.10.3) (43). We examined the expression of macrophage and T cell markers across clusters and identified a cluster exhibiting co-expression of both, designated as the Mac_T cluster. To ensure that this identification was not biased by apoptosis-related groups, we repeated the analysis on a subset of samples excluding apoptosis groups, where we again identified the same co-expression cluster. DEG analysis was conducted to compare the Mac_T cluster with macrophage and lymphocyte clusters. DEGs with log_2_FC > 0.25 and an adjusted p-value < 0.05 were selected for pathway analysis using the compareCluster function from the clusterProfiler package. GO-BP annotations were used for enrichment analysis.

### Differential state analysis using pseudobulk method

Differential state analysis in this study was performed using the muscat R package (version 1.16) (44). Starting from a Seurat object, we converted the data into a SingleCellExperiment format using the as.SingleCellExperiment function in Seurat. Single-cell data were then aggregated by clusters and samples using the aggregateData function in muscat for pseudobulk analysis. Differential expression analysis was conducted using a linear model with contrast information for two conditions. For the comparison between acute-infected and uninfected conditions, the uninfected group was used as the reference. Similarly, for the comparison between non-apoptosis and no-cocultivation groups, the no-cocultivation group served as the reference. Pseudobulk differential expression analysis was performed using edgeR (45). Genes with an adjusted p-value < 0.05 and an absolute log_2_FC > 0.25 were considered significantly differentially expressed. For the acute-infected vs. uninfected comparison, DEGs were further filtered to include only those expressed in at least 10% of cells in one or more groups. Visualization was carried out using built-in functions in muscat, and a multi-dimensional scaling (MDS) plot was generated using the pbMDS function.

### Statistics

DEG analysis at the single-cell level was performed using the non-parametric Wilcoxon rank-sum test, with p-values adjusted using Bonferroni correction. In most DEG analyses, significance was defined as an adjusted p-value < 0.05 and log_2_FC > 0.25. However, in certain comparisons, a higher log_2_FC threshold was applied, as specified in the Figure legends. For sample-level DEG or differential state (DS) analysis, edgeR or DESeq2 was used with specification in method and Figure legend. Similar to the single-cell analysis, genes with an adjusted p-value < 0.05 were considered significant. Pathway enrichment analysis was performed using a hypergeometric test, with p-values adjusted for multiple comparisons across clusters or conditions. An adjusted p-value < 0.05 was considered statistically significant.

## Results

### Characterization of a cell cluster having co-expression of myeloid and lymphoid genes in scRNA-seq.

To examine the lymphocyte population in the brain during the initial phase of SIV infection, we isolated enriched immune cells from three acutely infected (day 12 p.i.) rhesus macaques and three uninfected animals, and performed scRNA-seq. Following bioinformatic processing, we applied the initial clustering algorithm on 181,543 brain cells, resulting in 12 clusters. Then, we characterized each of those cell clusters by screening different leukocyte markers. Most cells were identified as microglia **(Suppl. Figure 1A)**. Additionally, four lymphocyte clusters with high lymphocyte marker expression **(Suppl. Figure 1B)** were identified, containing 26,340 cells. Except for two uncharacterized cell clusters, the other cell clusters in our brain dataset could be identified as microglia, lymphocytes, or CNS-associated macrophages (CAMs) **(Suppl. Figure 1C)**.

Interestingly, one of those cell clusters (i.e. cluster 8) had co-expression of myeloid and lymphoid cell markers, as well as a higher UMI count compared to microglia and lymphocyte clusters **(Suppl. Figure 1D)**. This could be, for example, cells transitioning between states, or perhaps a myeloid cell fusing with or phagocytosing a lymphoid cell. Another possibility is that these are artifacts due to an initial capture of two cells (thus a doublet) instead of a single cell in the initial stage of the scRNA-seq procedure, either because the cells are highly adherent to each other or due to an artefactual capture of two independent cells. To help assess this we applied DoubletFinder (36), a frequently used algorithm to identify doublets. This revealed that about half of the cells in cluster 8 were identified as doublets **(Suppl. Figure 1E)**. However, DoubletFinder is predicting doublets based on combining the transcriptional characteristics of pairs of cells within the dataset. Thus, it is difficult to distinguish the above possibilities. Therefore, we designed a study to validate this special observation on scRNA-seq experiment.

**Figure 1:**
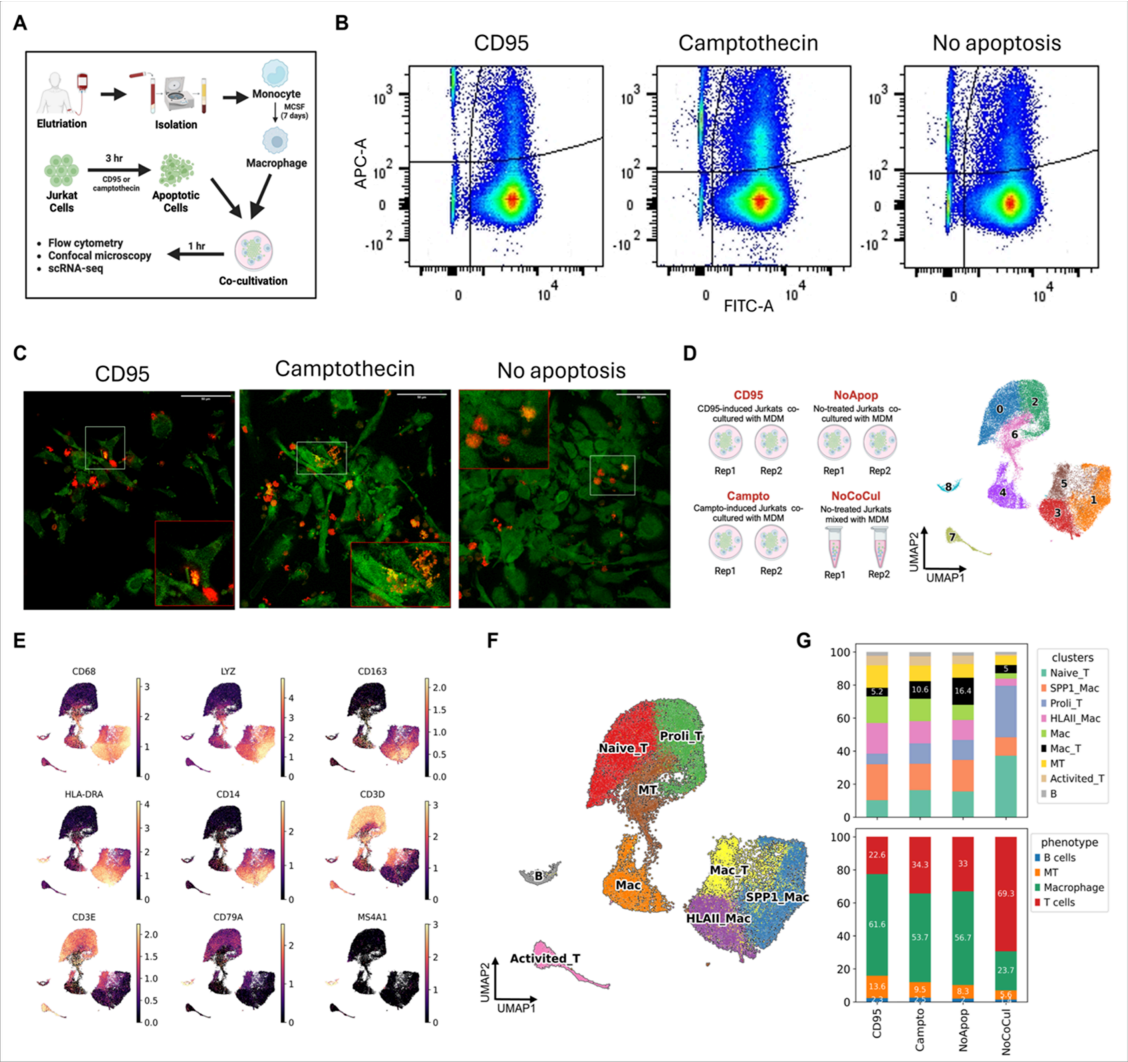
Identification of macrophage-T-cell coexistence cells (Mac_T). **(A)** Workflow for generating monocyte-derived macrophages (MDM) and Jurkat cells cocultivation system. **(B)** Flow cytometry showing the Mac_T cells in three treatment groups (i.e. CD95, Camptothecin and No Apoptosis groups). **(C)** Representative figures showing macrophage-T-cell coexistence cells for three treatment groups. For both flow cytometry and confocal microscopy experiments, the MDMs were labeled with CellTracker Green and Jurkat cells were labeled with CellTracker Deep Red before the cocultivation. **(D)** From scRNA-seq experiment for eight samples in four different groups (i.e. CD95, NoApop, Campto, and NoCoCul), the cells kept after quality control were clustered into nine different clusters. **(E)** The UMAP plot of the normalized expression of macrophage, T cell and B cell markers. **(F)** The UMAP plot of 42,390 cells within nine different clusters. The annotation for each cluster was labeled. **(G)** The proportion of each cluster (upper panel) or phenotype (lower panel) in each experimental group. Campto: camptothecin-treated MDM-Jurkat cocultivation group. CD95: CD95-treated MDM-Jurkat cocultivation group. NoApop: non-apoptosis MDM-Jurkat cocultivation group. NoCoCul: non-cocultivation MDM-Jurkat cocultivation group.

The experimental workflow is illustrated in Fig. 1A. We initially hypothesized that macrophages preferentially engulf apoptotic cells via efferocytosis. In this effort, we induced apoptosis in a human T-cell line (Jurkat cells) using biological and chemical agents prior to cocultivation with monocyte-derived macrophages (MDMs). Specifically, we evaluated the efficacy of Camptothecin (Campto) and anti-CD95 in inducing apoptosis in Jurkat cells. We found that both agents significantly increased apoptosis compared to the control, with anti-CD95 demonstrating greater potency **(Suppl. Figure 2A and** 2B). Extending the treatment from three to six hours increased the proportion of apoptosis in the Jurkat cells from 16 to 32% with Campto and from 22 to 62% with anti-CD95, however, the rate of apoptosis for untreated cells remained at 4%. After confirming the effectiveness of these apoptotic agents, we cocultured the Jurkat cells with MDMs. To facilitate tracking via flow cytometry and microscopy, MDMs and Jurkats were labeled with CellTracker dyes prior to cocultivation. Given the size differences between macrophages and T cells, the two populations were readily distinguishable based on forward and side scatter properties in flow cytometry. Double-positive cells, indicative of phagocytosis, were detected exclusively within the macrophage population and not in T cells **(Suppl. Figure 2C)**. Interestingly, double-positive cells were observed in both apoptotic and non-apoptotic (control) groups (Fig. 1B and S2D), suggesting that non-apoptotic T cells could also be efficiently phagocytosed by MDMs. This observation was further corroborated by confocal microscopy (Fig. 1C **and Suppl. Video.1**), which showed colocalization of MDMs and Jurkat cells in both apoptotic **(Suppl. Video. 1a and 1b)** and non-apoptotic conditions **(Suppl. Video. 1c)**, consistent with our flow cytometry findings.

Then we confirmed those phagocytosed cells on scRNA-seq. Cells from duplicate samples across four experimental conditions, including CD95-treated apoptotic, Campto-treated apoptotic, non-apoptotic, and a no-cocultivation control, were aggregated and clustered into nine cell clusters (Fig. 1D). Characterization revealed that clusters 1, 3, and 4 expressed macrophage markers, while clusters 0, 2, and 7 expressed T-cell markers. Cluster 6 was enriched for mitochondrial gene expression, cluster 8 expressed B-cell markers (likely due to contamination during monocyte isolation), and cluster 5 co-expressed macrophage and T-cell markers (Fig. 1E **and Suppl. Table. 3**). Within the macrophage subsets, cluster 1 was enriched for SPP1 and lysosomal genes, while cluster 3 exhibited high expression of HLA class II molecules. Among the T-cell subsets, cluster 2 showed elevated expression of proliferation markers (e.g. MKI67, TOP2A), whereas cluster 7 upregulated activation/differentiation markers associated with CD4 + T cells (e.g. CCL5, IL32, RORA) **(Suppl. Figure 2E and Suppl. Table. 3)**. Clusters 0 and 4 lacked distinct activation markers, suggesting they represented resting T cells and macrophages, respectively. Based on these characterizations, we annotated each cell cluster showed in Fig. 1F and quantified the relative proportions of each cluster across treatment conditions (Fig. 1G). Notably, the non-apoptotic group exhibited the highest proportion (~ 16.4%) of the macrophage-T-cell co-expressing cluster (Mac_T), followed by the Campto-treated apoptotic group (~ 10.6%). In contrast, the CD95-treated and no-cocultivation groups displayed the lowest percentage (~ 5%). These results suggest that the reduced presence of Mac_T cells in the CD95-treated group may be attributed to T-cell degradation. Indeed, this group exhibited the lowest overall T-cell abundance and the highest proportion of the MT cluster (Fig. 1G). Furthermore, the flow cytometry shown in **Suppl. Figure 2A** also suggested that CD95 had a greater ability to induce apoptosis in T cells compared to Campto. Nevertheless, the Mac_T cluster was consistently more abundant in all cocultivation conditions compared to the no-cocultivation control, indicating that scRNA-seq successfully captured macrophage-phagocytosed or efferocytosed T cells. To rule out potential biases introduced by CD95 or Campto treatment, we reanalyzed the non-apoptotic and no-cocultivation samples **(Suppl. Figure 2F)**. Similarly, a distinct cluster of co-expressing macrophage and T-cell markers was identified **(Suppl. Figure 2G)**, with its relative proportion being approximately threefold higher in the non-apoptotic cocultivation group **(Suppl. Figure 2H)**.

### Chemokine genes might serve as potential markers for identifying macrophage-T-cell co-expressing cells in scRNA-seq.

**Figure 2:**
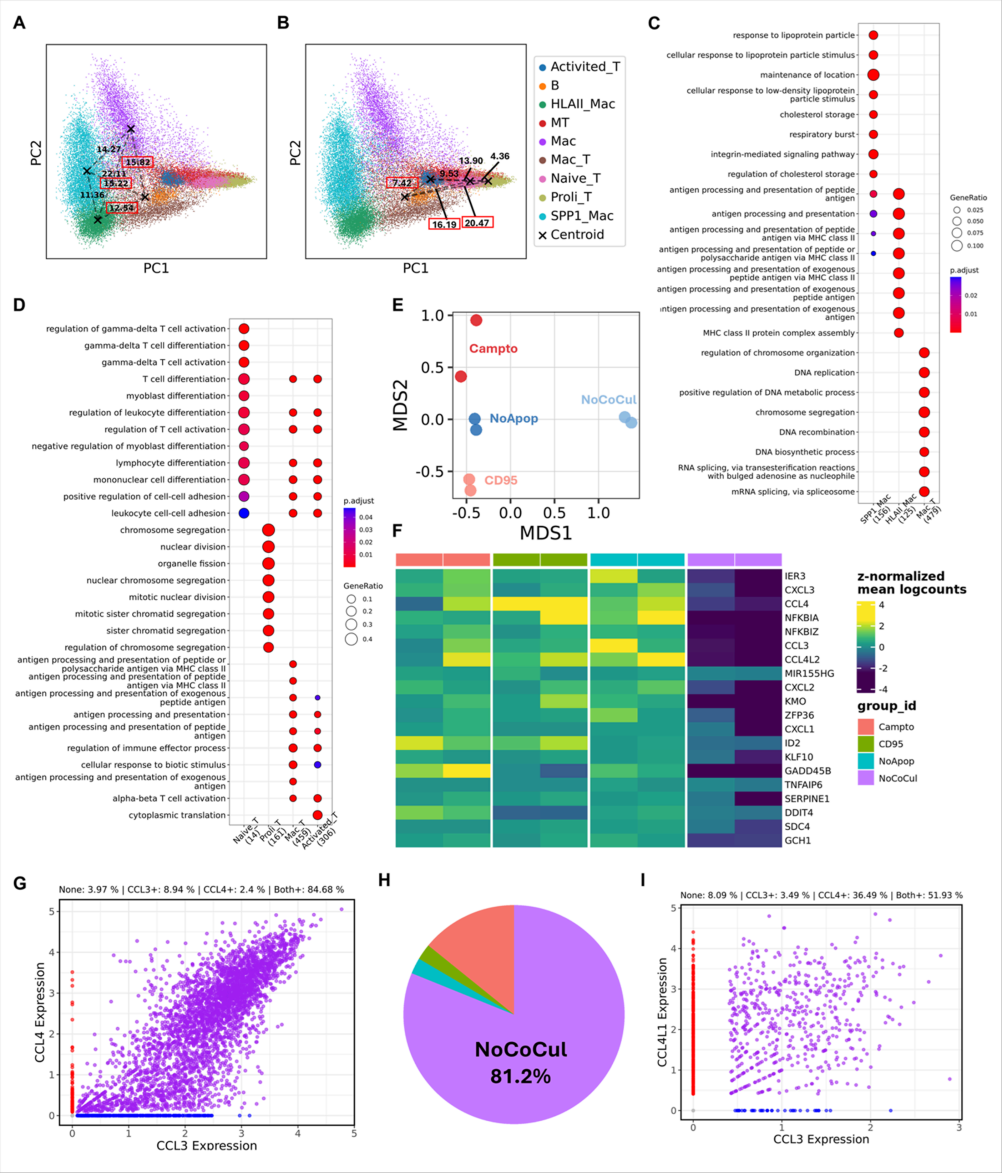
Characterizations of Mac_T cluster. **(A-B)** The distribution of 42,390 cells on the first two principal components (PCs). The cells were colored by the cluster information, and the Euclidean distance between Mac_T cluster and (A) other macrophage clusters or **(B)**lymphocyte clusters were labeled and highlighted. **(C)** Comparison of gene ontology pathways (Biological Process, BP) between SPP1_Mac, HLAII_Mac, and Mac_T clusters. **(D)** Comparison of gene ontology pathways (Biological Process, BP) between Naïve_T, Proli_T, Activated_T and Mac_T clusters. **(E)** Multidimensional scaling (MDS) plot displaying Mac_T cells in 8 samples from four different experimental groups. The expression of Mac_T cells were averaged in each sample for pseudobulk analysis. **(F)** Heatmap plotting of top 20 (ranked by adjusted p value) genes in Mac_T cluster that were significantly changed in NoApop group compared to NoCoCul group. The pseudobulk comparison was performed between two animals in NoApop and NoCoCul groups using EdgeR method. The genes with adjusted p-value < 0.05 and logFC > 0.25 were defined as significantly changed genes. **(G)** The scatter plot of the cells in Mac_T cluster regarding their expression of CCL3 and CCL4. **(H)** The proportion of each treatment group for CCL3 and CCL4 double negative cells in Mac_T cluster. **(I)** The scatter plot of the cells in the cluster of the rhesus macaque brain with co-expression of myeloid and lymphoid genes (Cluster 8 in **Figure S1C**) regarding their expression of CCL3 and CCL4L1. Campto: camptothecin-treated MDM-Jurkat cocultivation group. CD95: CD95-treated MDM-Jurkat cocultivation group. NoApop: non-apoptosis MDM-Jurkat cocultivation group. NoCoCul: non-cocultivation MDM-Jurkat cocultivation group.

In our characterization, we identified a Mac_T cell cluster with high expression of chemokines such as CCL2 and CCL3, which were also strongly expressed in HLAII_Mac but not in SPP1_Mac or general Mac clusters **(Suppl. Figure 2E)**. This suggests a distinct macrophage phenotype that preferentially engulfs Jurkat cells. To further investigate this, we computed the Euclidean distance between Mac_T and other macrophage clusters in a low-dimensional space. Notably, HLAII_Mac exhibited the shortest Euclidean distance to Mac_T compared to the other two macrophage clusters (Fig. 2A), indicating that HLAII-expressing macrophages may preferentially engulf Jurkat cells. Similarly, to identify specific T cell phenotypes preferentially engulfed by macrophages, we calculated the Euclidean distance between Mac_T and other T cell clusters. Mac_T was closest to the activated T cell cluster (Fig. 2B), suggesting that the T cell component within Mac_T originates might be primarily from activated T cells. Further gene ontology (GO) analysis revealed that macrophages and T cells within the Mac_T cluster exhibited upregulated pathways related to DNA replication, mRNA splicing, and MHC class II antigen processing and presentation (Fig. 2C and 2D, **Suppl. Table. 4**), indicating a unique immune state within this cluster.

Both Mac_T cells and technical doublets (macrophage-T cell aggregates from scRNA-seq artifacts) displayed co-expression of myeloid and lymphoid genes, leading to their clustering together. However, Mac_T cells likely undergo biological processes such as phagocytosis and efferocytosis, which are absent in technical doublets. Supporting this, the gene expression profiles of Mac_T cells differed significantly between cocultivation and no-cocultivation groups in low-dimensional space (Fig. 2E). To identify key genes driving these differences within the Mac_T cluster, we performed a pseudobulk analysis comparing Mac_T cells from the non-apoptotic and no-cocultivation groups. Notably, chemokine genes such as CXCL3, CCL3, CCL4, CCL4L2, and CXCL2 were significantly upregulated not only in the non-apoptotic group but also in the CD95 and Campto groups compared to the no-cocultivation group (Fig. 2F **and Suppl. Table. 5**). These findings suggest that chemokine genes may serve as markers to distinguish Mac_T cells from technical doublets. To further validate this, we examined the expression of CCL3 and CCL4, two chemokines highly expressed in both human MDM and rhesus macaque brain myeloid cells. Within the Mac_T cluster, approximately 85% of cells co-expressed CCL3 and CCL4, while only ~ 4% lacked expression of both genes (Fig. 2G). Notably, most of these CCL3 and CCL4 double-negative cells originated from the no-cocultivation group (Fig. 2H). Similarly, in co-expression cluster from rhesus macaque sample, only ~ 8% of cells were CCL3 and CCL4 double-negative (Fig. 2I). This indicates that the majority of cells in this cluster are likely biologically relevant Mac_T cells, rather than technical doublets.

### Acute SIV infection depletes CD4 + lymphocytes and promotes the expansion of proliferating cytotoxic T cells in the brain.

**Figure 3:**
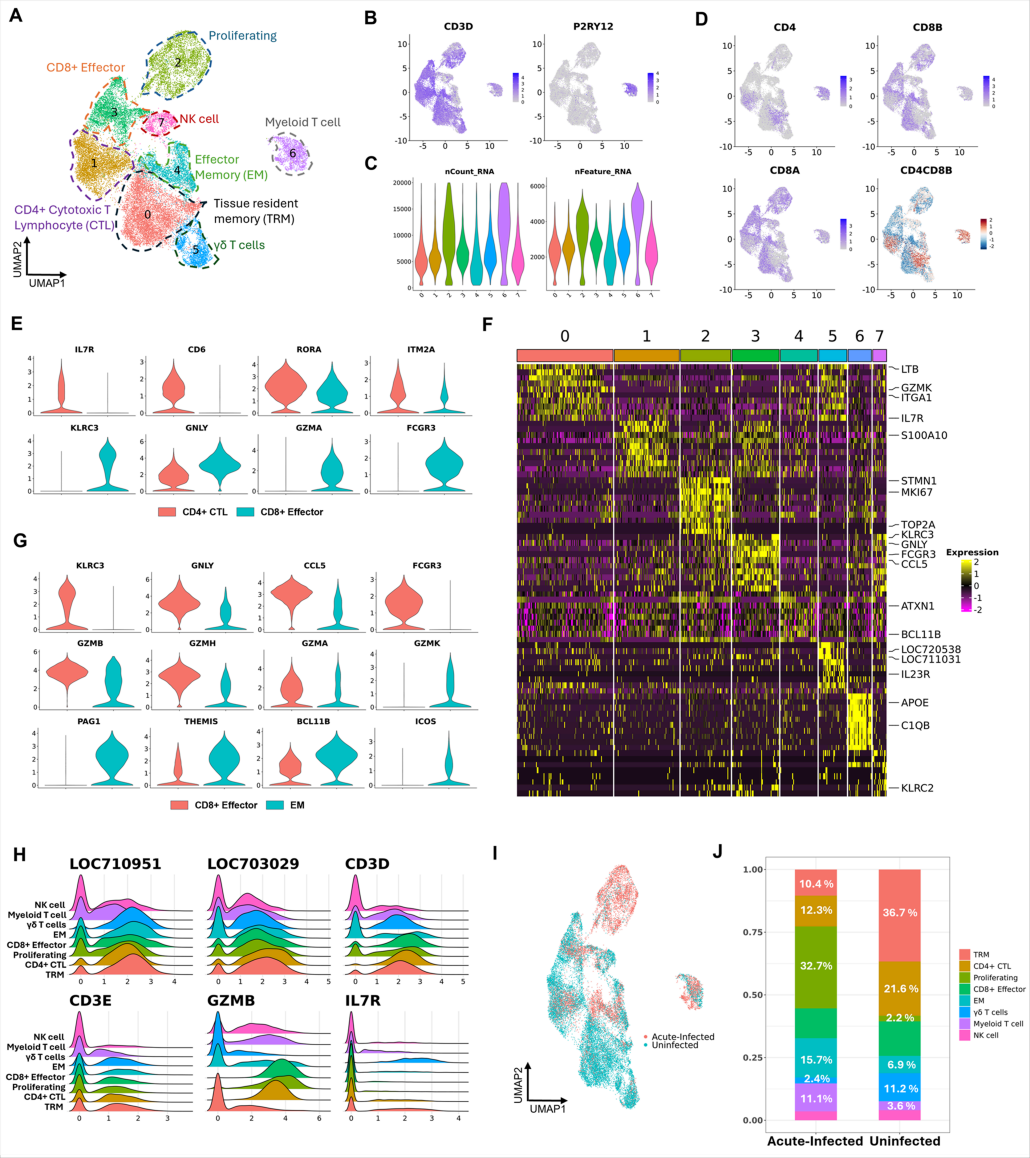
Characterization of brain lymphocytes in uninfected and acute-infected rhesus macaques. **(A)** The UAMP projection of 26,349 brain lymphocytes from infected and uninfected brains. The annotation for each brain lymphocyte cluster was labeled. **(B)** UMAP projection of normalized RNA expression of CD3D and P2RY12 genes. **(C)** Violin plots for UMI counts and gene counts for each brain lymphocyte cluster. **(D)** UMAP projection of normalized RNA expression of CD4, CD8A, CD8B genes and CD4 to CD8B ratio. **(E)** Violin plots for representative markers detected between CD4+ CTLs and CD8+ effector cells in the brain. **(F)** Heatmap for top 10 markers arranged by log_2_FC in each brain lymphocyte cluster. The scaled data was used for this plotting. **(G)** Violin plots for representative markers detected between CD8+ effector cells and effector memory (EM) cells in the brain. **(H)** Ridge plots for known markers for lymphocytes. **(I)** The UAMP projection of brain lymphocytes was labeled by infected status. **(J)** The relative proportion of each identified cluster in uninfected and acute-infected brain lymphocytes. The percentage of primarily changed cell clusters in uninfected and acute-infected conditions were labeled. The statistical test for marker detection was the Wilcoxon rank sum test with parameters as adjusted p-value < 0.05 and log_2_FC > 0.5. LOC710951: TCR α chain. LOC703029: TCR β chain. LOC720538: TCR γ chain. LOC711031: TCR δ chain.

The 26,340 cells from lymphocyte clusters and co-expression cluster (cluster 8) were re-clustered into eight different cell clusters, and the co-expression cluster (Myeloid-T cell cluster) separated from the other seven clusters from UMAP (Fig. 3A and 3B). This Myeloid-T cell cluster was also found with higher UMI, and gene counts than other clusters (Fig. 3C).

Based on their gene expression profiles, we characterized the other lymphocyte phenotypes in the brain. We found that the brain lymphocytes generally had lower expression of the CD4 gene compared to the CD8 genes. Single cell studies of rhesus monkey lymphocytes indeed find low expression of the CD4 mRNA. By calculating the log_2_ normalized CD4 to CD8B expression ratio for each single cell (Fig. 3D), we found that the cells in cluster 1 and part of cells in cluster 0 had high CD4 to CD8B ratio, indicating they likely represent CD4 + T cells in the brain. The cells in cluster 3 and another part of cells in cluster 0 had a negative CD4 to CD8B ratio, indicating likely correspond to CD8 + T cells. Finally, we classified the brain lymphoid cells as Tissue Resident Memory (TRM) cells, γδ T cells, Effector Memory (EM) cells, CD4 + cytotoxic T lymphocytes (CTLs), NK cells, CD8 + Effector cells, and proliferating cells (Fig. 3A). Regarding CD4 + CTLs, they have been found to be highly enriched in the subset of CD4 + effector memory T cells expressing CD45RA (46). An analysis assessing scRNA-seq data from FACS-purified T cells has characterized analogous cells as CD4 + effector memory T cells (47), and others have noted transcriptional similarities between CD4 + CTL and CD4 + effector memory T cells (46, 48). We will use the CD4 + CTL nomenclature here.

Many lymphocytes in the brain exhibited gene expression profiles indicating cytotoxic properties, which included cluster 1 (CD4 + CTL) and cluster 3 (CD8 + Effector). Therefore, the lymphocytes in those two clusters were all highly activated. However, there were some differences between them. For example, CD8 + effector cells were more NK-like, expressing NK cell markers (e.g. KLRC3, GNLY, FCGR3) not seen in CD4 + CTL (Fig. 3E). Instead, CD4 + CTL maintained IL7R expression, which should be downregulated following T cell activation (49). Additionally, compared to CD8 + effector cells in the brain, the CD4 + CTLs also had higher expression RORA, the marker for CD4 + Th17 cells (50), and the CD6 gene, an essential molecule for T cell-antigen presenting cell (APC) interaction (Fig. 3E) (51, 52). Cluster 0, which contained both CD4 + and CD8 + lymphocytes, was non-cytotoxic, and its detected markers contained CD69 and CD49a (ITGA1), which are known TRM T cell markers (Fig. 3F **and Suppl. Table. 6**) (53–55).

Distinguishing CD4 + and CD8 + lymphocytes in cluster 2 and cluster 4 was difficult since there were many double-negative cells, which could be caused by the sparsity of the data or their biological nature. However, more lymphocytes in those two cell clusters had a negative log_2_ CD4 to CD8B ratio (Fig. 3D). Given the uncertainty for the rest of the lymphocytes’ expression of CD4 and CD8, we annotated cluster 2 and cluster 4 by their predicted biological properties. For example, cluster 2 had a high expression of proliferating markers (i.e. STMN1, MKI67, TOP2A), so we annotated it as a proliferating cluster. Cluster 4 had an expression of the genes related to TCR stimulation and lymphocyte cytotoxicity (Fig. 3F **and Suppl. Table. 6**). However, compared to CD8 + effector cells, cluster 4 had higher GZMK expression but lower GZMA and GZMB expression (Fig. 3G), consistent with the granzyme gene changes during CD8 + memory T cell differentiation (56). Therefore, the evidence from scRNA-seq data indicated that cluster 4 might be effector memory (EM) lymphocytes.

Unlike lymphocyte clusters mentioned above, all the cells in clusters 5 and 7 barely expressed CD4 and CD8B (Fig. 3D), suggesting they were double negative cells. Cluster 7 expressed cytotoxic genes and some NK cell markers and had the lowest expression of TCR αβ chains (e.g. LOC710951 and LOC703029) and CD3 δε chains compared to other T cell clusters, so was classified as NK cells. Cluster 5 had expressions of TCR gamma and delta chains (i.e. LOC720538 and LOC711031) and other γδ T17 cell markers, including IL23R and CCR6 (Fig. 3F). In addition, the CD69 and CD103 (ITGAE) playing a crucial role in lymphocytes’ initial tissue retention and prolonged residence (57) were also found to be highly expressed in γδ T cells, which is consistent with the fact that the meningeal γδ T cells are thought as tissue-resident cells in the brain (58). Interestingly, most cells in γδ T cluster (cluster 7) had RNA expression of TCR αβ chains as well as γδ chains (Fig. 3H). Most of our manual annotations based on well-known cell markers were further confirmed by one of the cell type annotation tools, Single-cell Mayo Map (scMayoMap) (38) **(Suppl. Figure 1F)**.

Most brain lymphocytes highly expressed cytotoxic genes and only the TRM and γδ T clusters of cells had low expression of cytotoxic genes (Fig. 3H). Interestingly, naïve T cell populations were not found in infected or uninfected brains, indicating that the brain lymphocytes are all in a differentiated state. The other clusters, comprising CD4 + CTL, CD8 + effector, proliferating T, CD8 + EM, Myeloid-T, and NK cells, expressed more cytotoxic genes. In response to acute SIV infection, the TRM (especially CD4+), CD4 + CTL, and γδ T cells were depleted in the brain relative to uninfected animals. Proliferating cytotoxic T cells, EM and Myeloid-T cell clusters increased, while the CD8 + effector and NK cell clusters barely changed in proportion at this time point (Fig. 3I and 3J). Among the three increased lymphocyte populations, the proliferating cluster had the largest increase (~ 15-fold).

### Acute SIV infection cause the depletion of CD4 + memory T cells and increasing of CD4 + CTLs in the peripheral blood.

**Figure 4:**
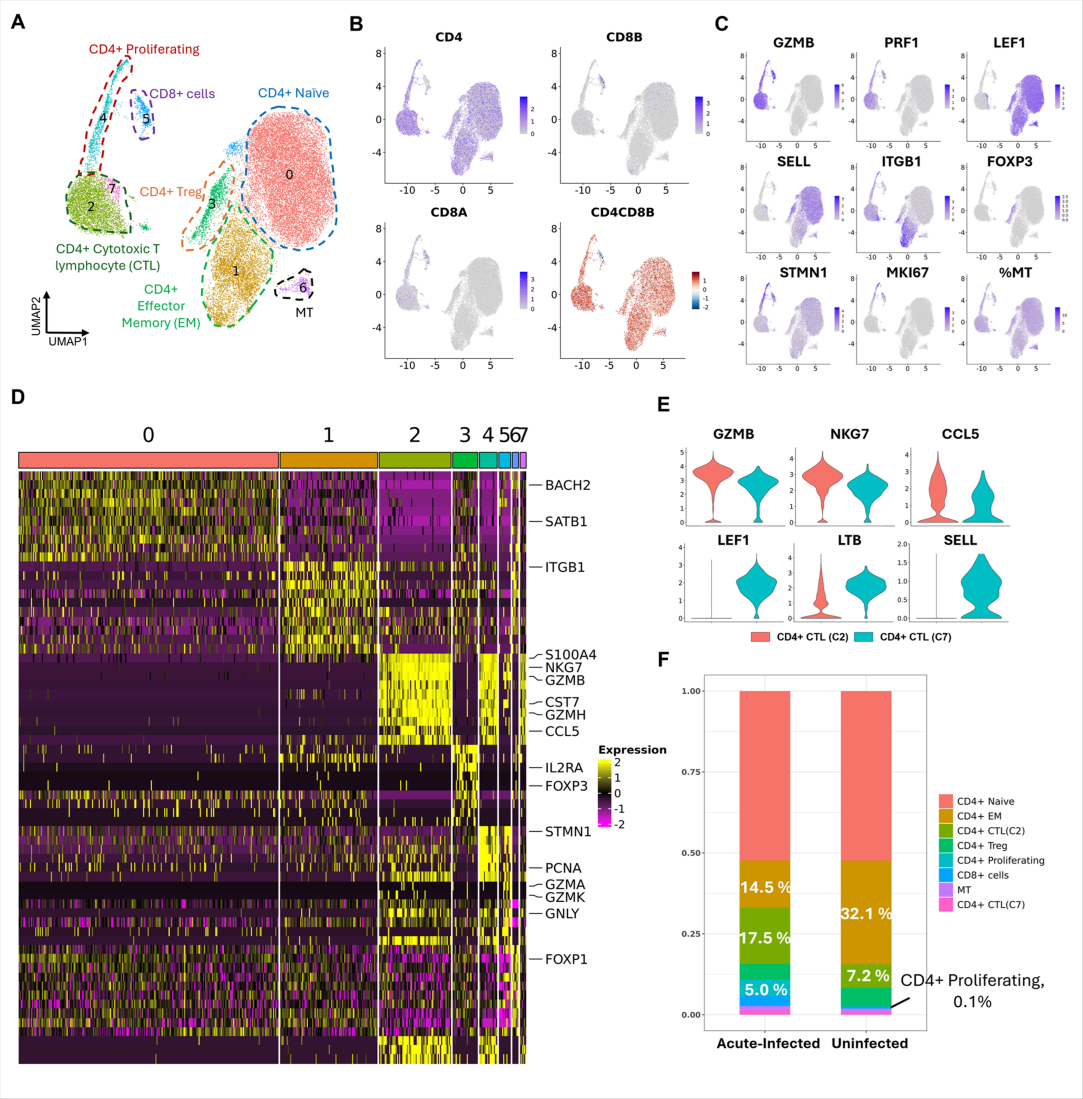
Characterization of T cells in CD4-sorted blood of uninfected and acute-infected rhesus macaques. **(A)** The UAMP projection of 25,808 CD4-sorted blood T cells from infected and uninfected animals. The annotation for each blood lymphocyte cluster was labeled. **(B)** UMAP projection of normalized RNA expression of CD4, CD8A, CD8B genes and CD4 to CD8B ratio. **(C)** UMAP projection of normalized RNA expression of known markers for different lymphocyte phenotypes and mitochondrial percentage (mitoPercent). **(D)** Heatmap for top 10 markers arranged by log_2_FC in each blood lymphocyte cluster. The scaled data was used for this plotting. **(E)** Violin plots for representative markers detected between two CD4+ CTL clusters in blood. **(F)** The relative proportion of each identified cluster in uninfected and acute-infected blood lymphocytes (CD4-sorted). The percentage of primarily changed cell clusters in uninfected and acute-infected conditions were labeled. The statistical test for marker detection was the Wilcoxon rank sum test with parameters as adjusted p-value < 0.05 and log_2_FC > 0.5.

Given that prior to entering the brain the CNS lymphocytes were blood-borne, we next studied blood lymphocytes. In the periphery, the main target for HIV/SIV is CD4 + T cells, so we focused on this cell type. Using freshly isolated PBMCs from the three acutely infected animals and one uninfected animal, CD4 + T-cells were FACS-purified and processed for scRNA-seq. The clustering on those CD4 + T-cell-sorted blood lymphocytes from the four animals generated eleven cell clusters **(Suppl. Figure 3A and Suppl. Figure 3B)**. Of the eleven cell clusters, eight expressed lymphocyte markers **(Suppl. Figure 3C)**, three expressed monocyte markers **(Suppl. Figure 3D)**, and none had co-expression of lymphoid and myeloid markers. We subset the eight lymphocyte clusters and reclustered the cells. The re-clustering generated eight different lymphocyte clusters (Fig. 4A). All eight clusters had high expression of the CD4 gene and a positive log_2_ CD4 to CD8B ratio (Fig. 4B). However, cells in cluster 5 exceptionally had a negative log_2_ CD4 to CD8B ratio, and high expression of cytotoxic genes, suggesting that this cluster contain contaminating CD8 + T cells. The transcriptomic profile of the cells in cluster 6 indicated their naïve or memory T cell nature. However, a higher proportion of mitochondria genes were found as markers in cluster 6, so this cluster might consist of naïve or memory T cells in a particular energetic state, denoted as MT. Besides clusters 5 and 6, the other five lymphocyte clusters were general CD4 + T cell phenotypes in the peripheral blood.

Most CD4 + blood lymphocytes were clustered into cluster 0, cluster 1, and cluster 3, which were non-cytotoxic T cells (Fig. 4C **and Suppl. Table. 7**). Cluster 0 was found with naïve T cell markers (e.g. LEF1 and SELL), cluster 1 was found with effector memory T cell markers (e.g. ITGB1 and ICOS), and cluster 3 was characterized by regulatory T cell (Treg) markers (e.g. IL2RA and FOXP3) (Fig. 4D). In contrast, cluster 2, cluster 4, and cluster 7 were CD4 + CTLs (Fig. 4C **and Suppl. Table. 7**), and markers for cluster 4 also included proliferating genes (STMN1, MKI67, TOP2A) (Fig. 4D). By comparing cluster 2 and cluster 7, we found that the cytotoxic genes were expressed at higher levels in cluster 2. In addition, cluster 2 completely lost the expression of naïve T cell genes, which still maintained expression in cluster 7 (Fig. 4E). Therefore, we hypothesized that cluster 7 might be in a transitional phase to become CD4 + CTLs. Those annotations were also confirmed by the cell type annotation tool, scMayoMap **(Suppl. Figure 3E)**.

During acute infection, we found that the CD4 + effector memory cells, were relatively decreased (from 32.1–14.5%) (Fig. 4F). Additionally, the percentage CD4 + Treg was also relatively decreased in acute infected animals, but with a small degree of change (from 5.8–4.8%). The decrease of CD4 + effector memory cells and Treg was accompanied by a dramatic increase of CD4 + CTLs. Those findings were consistent with the current report that the CD4 + naïve T cells might be less susceptible to HIV/SIV infection (9, 59, 60). In summary, the changes of CD4 + lymphocytes in the blood are partially consistent with what we found in the brain. The CD4 + memory T cells (EM in the blood and TRM in the brain) were the primary CD4 + lymphocytes depleted during the acute SIV infection in both peripheral and CNS.

### Brain lymphocytes undergo extensive activation during acute SIV Infection, with effector memory T Cells showing the strongest response.

**Figure 5:**
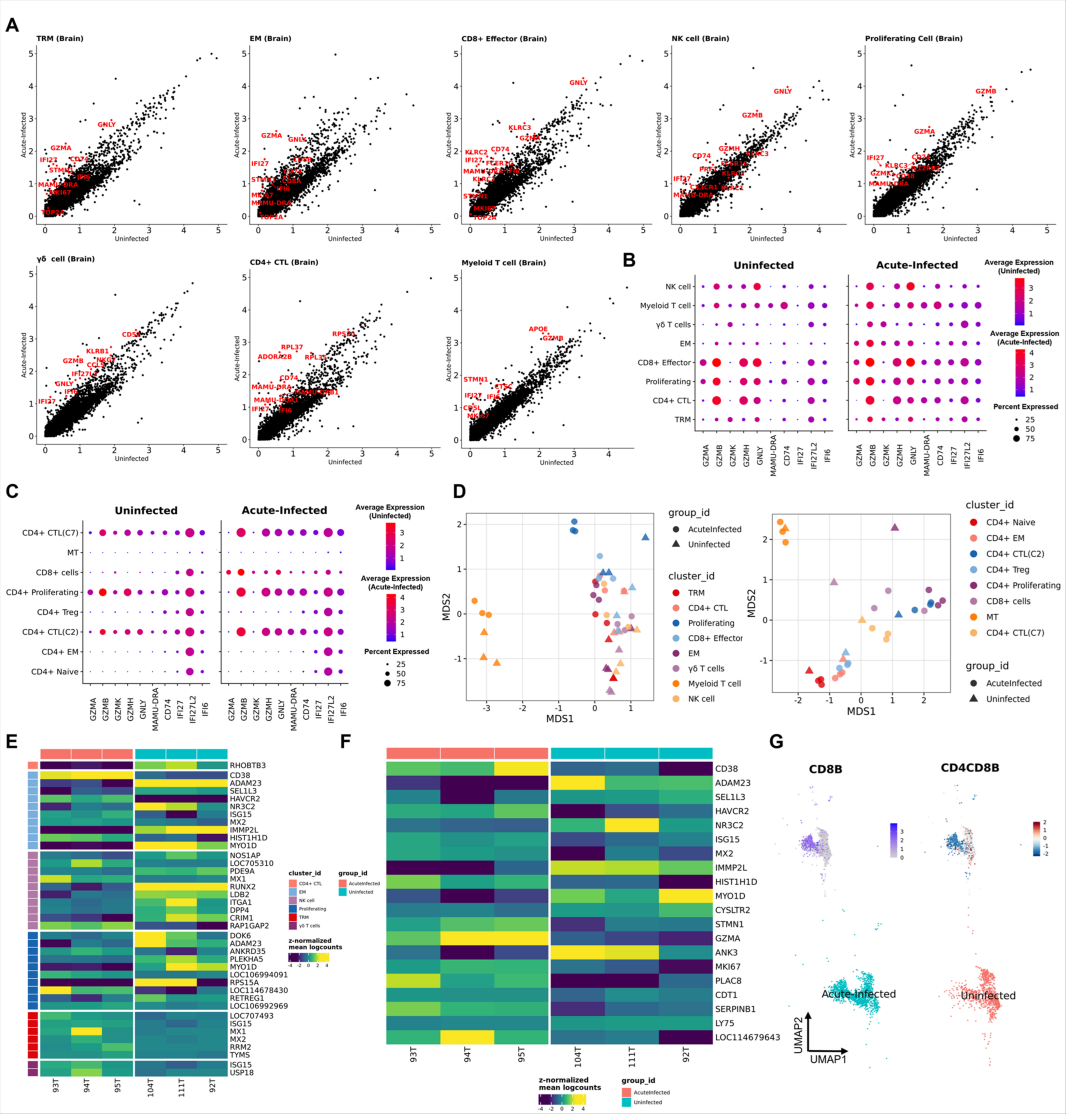
The transcriptomic changes of different brain lymphocyte phenotypes in response to acute SIV infection. **(A)** The gene scatter plot compared average gene expression in identified brain T cell and myeloid-T cell clusters between acute-infected and uninfected conditions. The statistical test for marker detection between lymphocytes found in infected and uninfected animals was the Wilcoxon rank sum test with parameters as adjusted p-value < 0.05 and log_2_FC > 0.5. The representative marker genes were labeled red in the gene scatter plots. **(B-C)** Comparison of the expression of selected cytotoxic genes, MHC class II molecules, and interferon-inducible proteins in different (B) brain and (C) blood lymphocyte clusters between uninfected and acute-infected conditions. **(D)** Multidimensional scaling (MDS) plot displayed brain (left) and blood (right) samples from acute and uninfected conditions. The plots were colored by cluster information. The expression of each lymphocyte cluster was averaged in each sample for pseudobulk analysis. **(E)** Heatmap plotting of top 10 (ranked by adjusted p value) genes in each brain lymphocyte cluster that were significantly changed in acute infection group compared to uninfected conditions. **(F)** Heatmap plotting of top 20 (ranked by adjusted p value) genes in brain EM cluster that were significantly changed in acute infection group compared to uninfected conditions. The pseudobulk comparison was performed between three acute-infected and uninfected animals using EdgeR method. The genes with adjusted p-value < 0.05 and logFC > 0.25 and expressed in an average of 10% of cells in at least 1 group were defined as significantly changed genes. **(G)** The UMAP projection of normalized CD8B expression and CD4 to CD8B ratio (upper panel), and the UMAP projection of cells from acute-infected and uninfected brains in EM cluster (lower panel).

We examined transcriptomic changes within individual lymphocyte clusters responding to acute SIV infection, with a specific focus on brain lymphocytes. We did comparisons using statistical methods for both single-cell and bulk data. For the comparison at single-cell level, we observed a significant upregulation of cytotoxic, MHC class II, and interferon-inducible genes during acute infection (Fig. 5A). Notably, while brain-resident CD4+ CTLs exhibited high baseline expression of cytotoxic genes, they did not further upregulate these genes in response to infection, unlike their counterparts in the blood **(Suppl. Figure 4A and Suppl. Figure 4B)**. This suggests a potential ceiling effect, as their cytotoxic gene expression was already elevated in uninfected brains. However, brain CD4+ CTLs did upregulate MHC class II molecules and interferon-related genes, indicating a heightened activation state. In contrast, upregulation of cytotoxic, MHC class II, and interferon-inducible genes was only observed in blood CD4+ CTLs **(Suppl. Figure 4C)** and not in other blood CD4+ T-cell clusters **(Suppl. Figure 4D)**. Further analysis of T-cell activation genes in brain (Fig. 5B) and blood (Fig. 5C) clusters showed that non-cytotoxic T cells in the brain acquired cytotoxicity during acute infection, whereas non-cytotoxic CD4+ T cells in the blood did not. This suggests that brain lymphocytes develop enhanced antiviral capabilities compared to those in the blood during acute SIV infection.

Multi-dimensional scaling (MDS) analysis at the sample level revealed a clear separation between acute-infected and uninfected animals along the second dimension (MDS2), indicating that acute infection affected multiple lymphocyte phenotypes in both the brain and blood (Fig. 5D).

Additionally, the Myeloid-T cluster was distinct from other brain lymphocyte clusters, while blood lymphocyte cluster with high mitochondria percentage (MT cluster), non-cytotoxic, and cytotoxic T-cell clusters also separated along MDS1, reflecting their transcriptomic differences.

To minimize the risk of inflated statistical significance caused by treating individual cells as independent replicates, we conducted pseudobulk comparisons between acutely infected (n = 3) and uninfected (n = 3) animals for each brain T-cell cluster by using the muscat implementation of edgeR. This comparison identified 522 significantly altered genes across different brain lymphocyte clusters **(Suppl. Table. 8)**. The EM cluster exhibited the highest number of significantly changed genes, followed by proliferating T cells and NK cells **(Suppl. Figure 4E)**. Among these genes, ISG15, an interferon-stimulated gene induced by type I interferons during viral infection, was significantly upregulated across multiple brain lymphocyte clusters, including tissue-resident memory (TRM), γδ T cells, proliferating T cells, and EM clusters (Fig. 5E **and Suppl. Table. 8**). Notably, ISG15 has been implicated in HAND and proposed as a potential biomarker for disease progression (61–63). Interestingly, USP18, a negative regulator of ISG15 signaling (64), was also significantly upregulated in some of these clusters (γδ T cells, proliferating T cells, and EM clusters). In the EM cluster, which exhibited the most substantial transcriptional changes during acute SIV infection, we observed significant upregulation of T-cell activation and proliferation genes such as CD38, GZMA, STMN1, and MKI67 in all acutely infected animals (Fig. 5F), indicating that this cluster was actively involved in immune activation and proliferation. Notably, CD8+ T cells within this cluster were predominantly derived from acutely infected animals (Fig. 5G), further highlighting the profound impact of acute SIV infection on brain EM T cell activation.

### Infected CD4 + cytotoxic T lymphocytes might be the primary lymphoid cells seeding the SIV in the CNS.

The infected CD4 + T cells in the bloodstream are one of the ‘Trojan horse’ candidates that can bring the HIV/SIV into the CNS during acute infection. However, different lymphocyte phenotypes might have different susceptibilities to HIV/SIV and varied chemotactic abilities, which determine their capabilities in seeding the virus in the brain.

**Figure 6:**
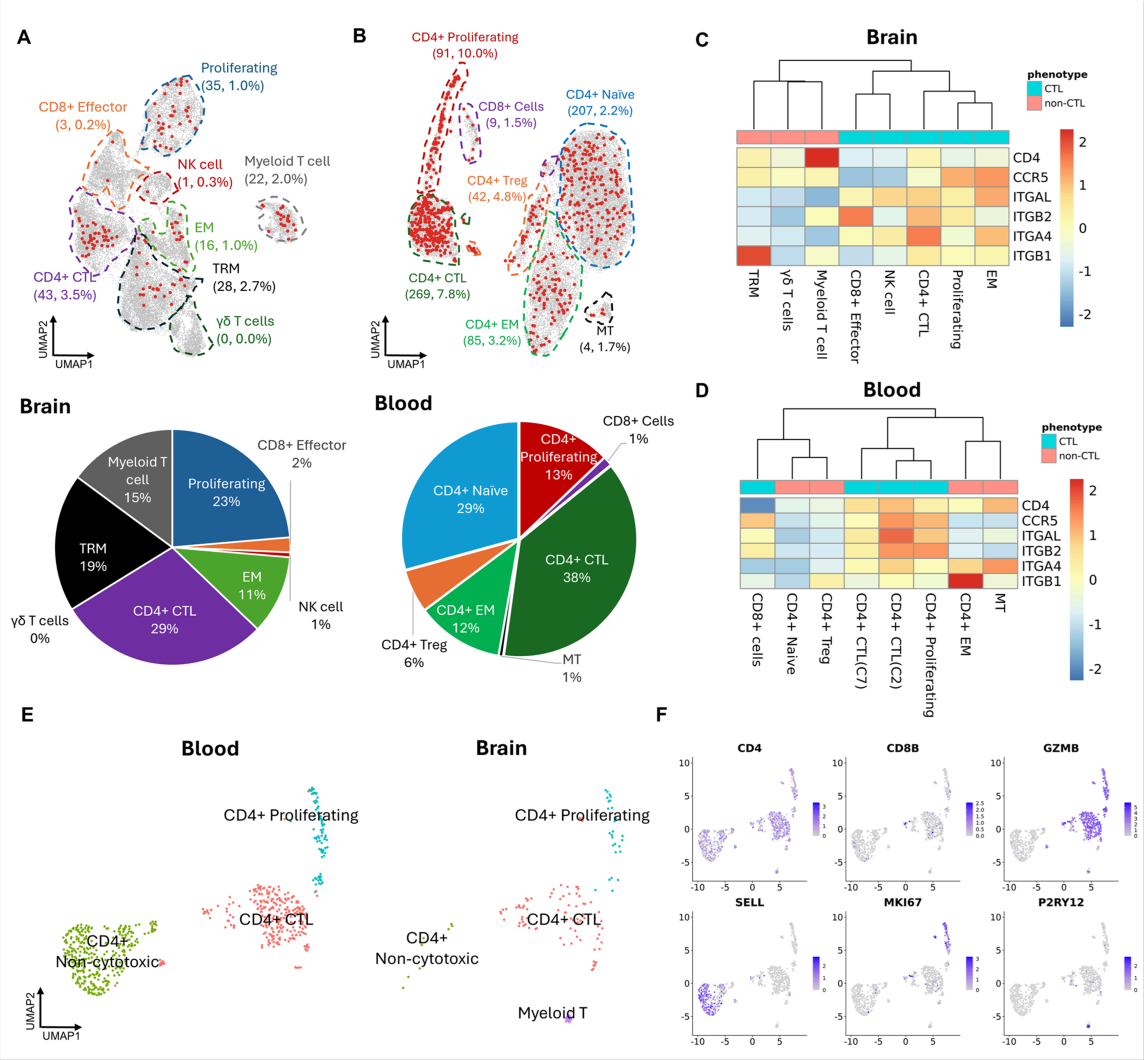
Characterization of SIV-infected cells in the brain and blood. **(A-B)** The UMAP projection of cells isolated from the brain and blood with the number and percentage of SIV+ cells in each brain/blood cell cluster (upper panel). The pie chart shows the relative contribution of each brain/blood cell cluster to the SIV+ cells (lower panel). **(C-D)** The heatmap for expression of genes that might contribute to the CNS infection (i.e. CD4, CCR5, ITGAL, ITGB2, ITGA4, ITGB1) in each brain/blood cell cluster. The scaled data was used for this plotting. **(E)** The UMAP projection for SIV-positive cells in blood and brain. **(F)** The UMAP projection of normalized RNA expression for known T cell, myeloid cell, and proliferating markers.

Among the lymphocyte clusters in the brain, the CD4 + CTL, TRM, and Myeloid-T cell clusters were the top 3 lymphocyte clusters with the highest proportion of SIV-infected cells. The percentage of SIV + cells in those three clusters was above or equal to 2%, and the SIV + cells in those three clusters contributed to 63% of SIV + cells in the brain (Fig. 6A). The CD8 + effector, NK, and γδ T cell clusters were the least infected as expected. For the blood lymphocytes, most of the infected cells were found in the CD4 + CTL clusters and thus comprised ~ 51% of the infected cells. While CD4 + Naïve cells comprised the second highest proportion of infected cells at 29%, they are an abundant cell type in the blood and the overall infection rate was much lower (2.2%) (Fig. 6B).

The infection rate in different lymphocyte clusters corresponded to their expression of CD4 and CCR5 (Fig. 6C **and** Fig. 6D). The average expression of CD4 in CD4 + CTL, TRM, and Myeloid-T cell clusters in the brain was higher than others, which might be one of the explanations for more SIV transcripts found in them. In contrast, the CD8 + effector, NK, and γδ T cell clusters in the brain had low to no expression of CD4 and CCR5, making them less susceptible to SIV (Fig. 6C). Similar to the CD4 + CTLs in the brain, this phenotype also had highest expression of CD4 and CCR5 compared to other CD4 + T cell clusters in the blood (Fig. 6D).

Based on above analyses, we hypothesized that CD4 + CTLs might be the primary CD4 + T cell phenotype seeding the SIV to the CNS, which was further confirmed by performing clustering only on SIV + cells in the brain and blood. Four clusters for SIV + cells, including CD4 + non-cytotoxic (includes CD4 + Naïve and CD4 + EM), CD4 + CTL, CD4 + proliferating, and Myeloid-T clusters were identified (Fig. 6E and 6F). The infected non-cytotoxic CD4 + T cells, which were abundant in the blood were barely found in the brain, confirming that CD4 + lymphocytes with cytotoxic properties were the primary lymphocyte phenotype seeding the SIV in the brain. The enhanced infiltrating ability of infected CD4 + CTLs could be attributed to their higher expression of the integrin alpha and beta chains for two adhesion molecules (LFA-1 and VLA-4) mainly expressed on lymphocytes compared to the other lymphocyte clusters (Fig. 6C and 6D). The naïve T cells in the blood barely expressed either of those two adhesion molecules, indicating their poor ability to travel to or from the CNS. This also helped explain why despite the abundance of this phenotype in the blood, none were found in the brain.

### The brain environment might promote the maturation of infiltrating lymphocytes from the bloodstream.

**Figure 7:**
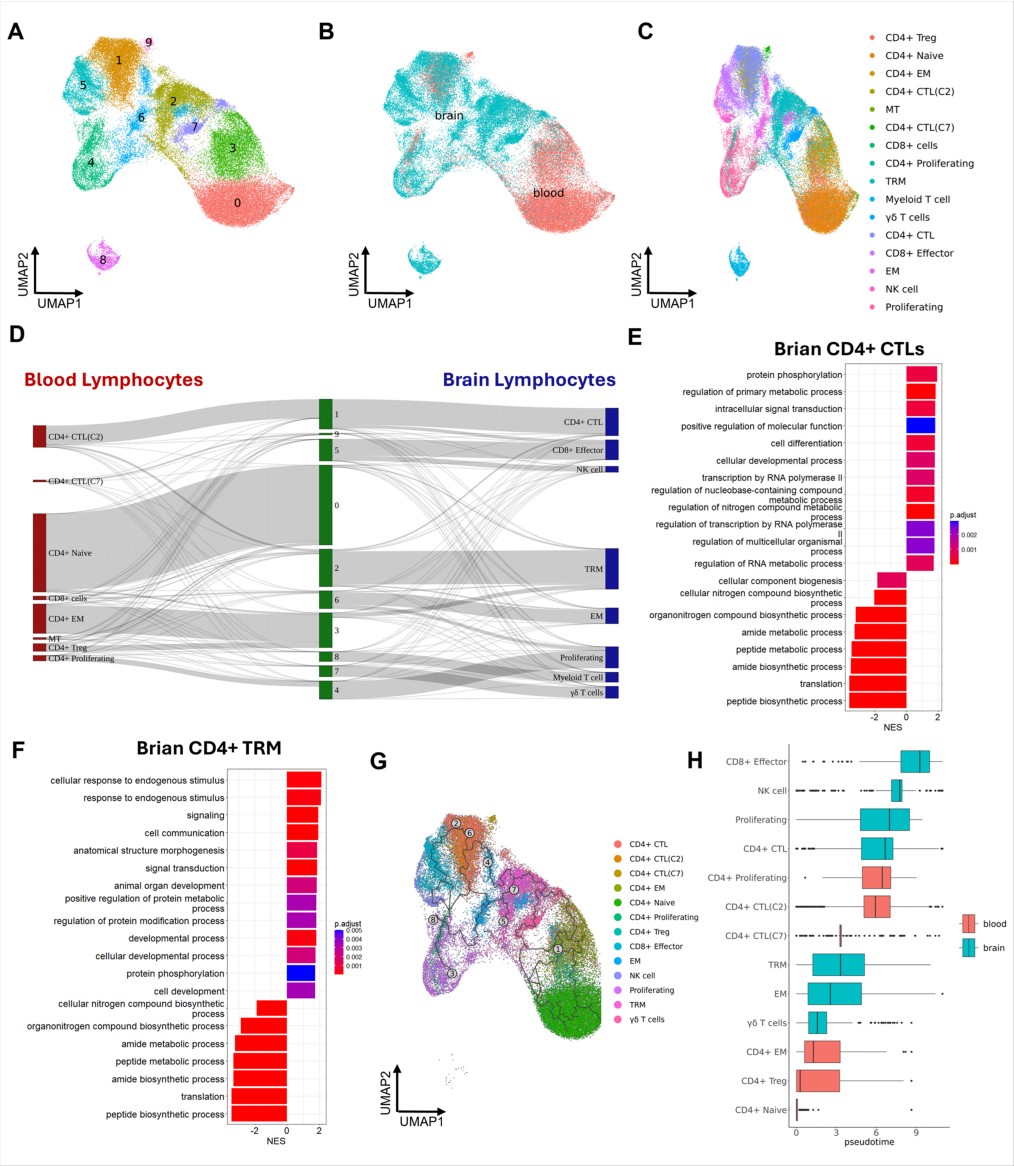
Characterization of connections between isolated blood and brain cells. **(A)** The UMAP projection for all 52,148 brain and blood cells. The cells are labeled by new cluster information by reclustering all 52,148 cells. **(B)** UMAP projection as in (A) labeled by the tissue they are isolated from. **(C)** UMAP projection as in (A) labeled by the previous cluster information generated by clustering on brain and blood cells separately. **(D)** The Sankey plot showed the connection between brain and blood lymphocyte phenotypes. The left (red) and right (blue) cluster information are generated by clustering separately for the blood and brain datasets. The cluster information in the middle (green) is through clustering on the merged dataset. The bar plot showed the top 20 upregulated and downregulated pathways enriched for the **(E)** brain CD4+ CTLs and **(F)**CD4+ TRMs. The Gene Set Enrichment Analysis (GSEA) was used for analyzing the altered pathways of the CD4+ CTLs and CD4+ TRM cells in the brain compared to CD4+ CTLs and CD4+ EM cells in the blood. The pathways with p.adjust < 0.05 were selected as enriched pathways and they were then ranked by normalized enrichment score (NES) for top 20 pathways. **(G)** The UMAP projection for all isolated brain and blood cells. The cells were colored by clusters generated by clustering on brain and blood cells separately. The grey circles labeled on the UMAP indicate the differentiation outcomes (cell fate) for setting the CD4+ Naïve T cell as the root node. **(H)** The box plot showing the predicted pseudo-time for cells in different clusters of brain and blood. The boxes were ordered by the median pseudo-time value for each cluster.

To understand the connections between the lymphocytes in peripheral blood and CNS. We aggregated all brain and blood lymphocytes isolated in this study and reperformed clustering, which generated 10 different cell clusters (Fig. 7A). Few overlaps between lymphocytes from brain and blood were found (Fig. 7B), which could be caused by most brain lymphocytes being CD8+ (65, 66). Still, we found that three clusters (i.e. clusters 1, 3, and 4) contained the T cells from both brain and blood. Cluster 1 contained 40% of cells from the CD4 + CTL(C2) cluster in the blood and 60% of cells from the CD4 + CTL cluster in the brain, indicating most CD4 + CTLs in the brain might be derived from the CD4 + CTL(C2) cluster in the blood. Most of the lymphocytes in cluster 3 were from CD4 + EM or CD4 + Treg in the blood, but ~ 12% of cells in this cluster were from the TRM cluster in the brain, which indicates the CD4 + EM and Treg might be the progenitor cells for the CD4 + TRM cells in the brain. Cluster 4 contained the proliferating CD4 + CTLs in the blood and the proliferating CD8+/CD4 + lymphocytes in the brain (Fig. 7C and 7D).

Aggregating the blood and brain lymphocytes for analyses further confirmed that CD4 + CTLs and CD4 + EM in the blood were the primary lymphocytes infiltrating the brain and raised the possibilities that the CD4 + EM might further differentiate into, or be replaced by, TRM cells in the brain. To understand how the brain environment changes the lymphocyte transcriptomic profiles, we performed DEG analyses for the brain and blood lymphocytes in those two clusters. We also found that once the CD4 + CTLs infiltrated from the blood to the brain, they downregulated the expression of many ribosomal genes and upregulated the transcription factors related to lymphocyte maturation/activation (e.g. FOS, FOSB, VAV3, ZEB2, KLRD1, and IFRD1) that regulate T cell activation, suggesting that the brain environment might reshape the CD4 + CTLs by affecting their gene regulations **(Suppl. Figure 5A)**. To ensure the DEG analysis was performed between CD4 + EM in the blood and CD4 + TRM in the brain, we first subset the cells with positive CD4 to CD8B ration in the brain TRM cluster. Then, we found that the downregulated genes for CD4 + EM in the blood also included a lot of ribosomal genes **(Suppl. Figure 5B)**. For the upregulated genes, it included the genes for lymphocyte cytotoxicity (e.g. CST7), TCR signaling (e.g. SKAP2), and lymphocyte motility (e.g. S100A4 and RGS1). The gene expression alterations aligned with observed pathway changes in both CD4 + CTLs and CD4 + TRM cells in the brain **(Suppl. Table. 9)**. Upon entering the brain and undergoing some differentiations, CD4 + CTLs and CD4 + TRM cells downregulated protein and peptide biosynthesis or metabolic pathways. Conversely, they upregulated pathways associated with intracellular signal transduction, protein phosphorylation, cell differentiation, and cellular development (Fig. 7E and 7F), reflecting increased cellular activity. These findings suggest that the brain environment reshaped the transcriptomic profile of blood’s CD4 + CTLs and CD4 + EM cells, promoting a more activated and mature state.

The trajectory analysis was performed to confirm the differentiation of the lymphocytes in the brain and blood. There were 8 differentiation outcomes (cell fate) found when we set the CD4 + naïve T cell cluster as the root node for predicting the differentiation path (Fig. 7G). The cell fates of CD4 + Naïve T cells included differentiating to CD4 + EM, CD4 + CTL, Proliferating, and TRM T cells. The pseudotime analysis revealed that the lymphocytes with cytotoxicity were more activated than all non-cytotoxic lymphocyte phenotypes in the brain or blood. Intriguingly, the brain lymphocyte phenotypes were more differentiated than blood lymphocyte phenotypes when we compared them within cytotoxic or non-cytotoxic lymphocyte populations (Fig. 7H). Therefore, pseudotime analyses further implied that the mature lymphocytes might be more differentiated when they migrate from the peripheral blood to the brain.

### 21 days of acute SIV infection led to more infected tissue resident memory T cells but less infected proliferating cytotoxic T cells in the brain.

**Figure 8:**
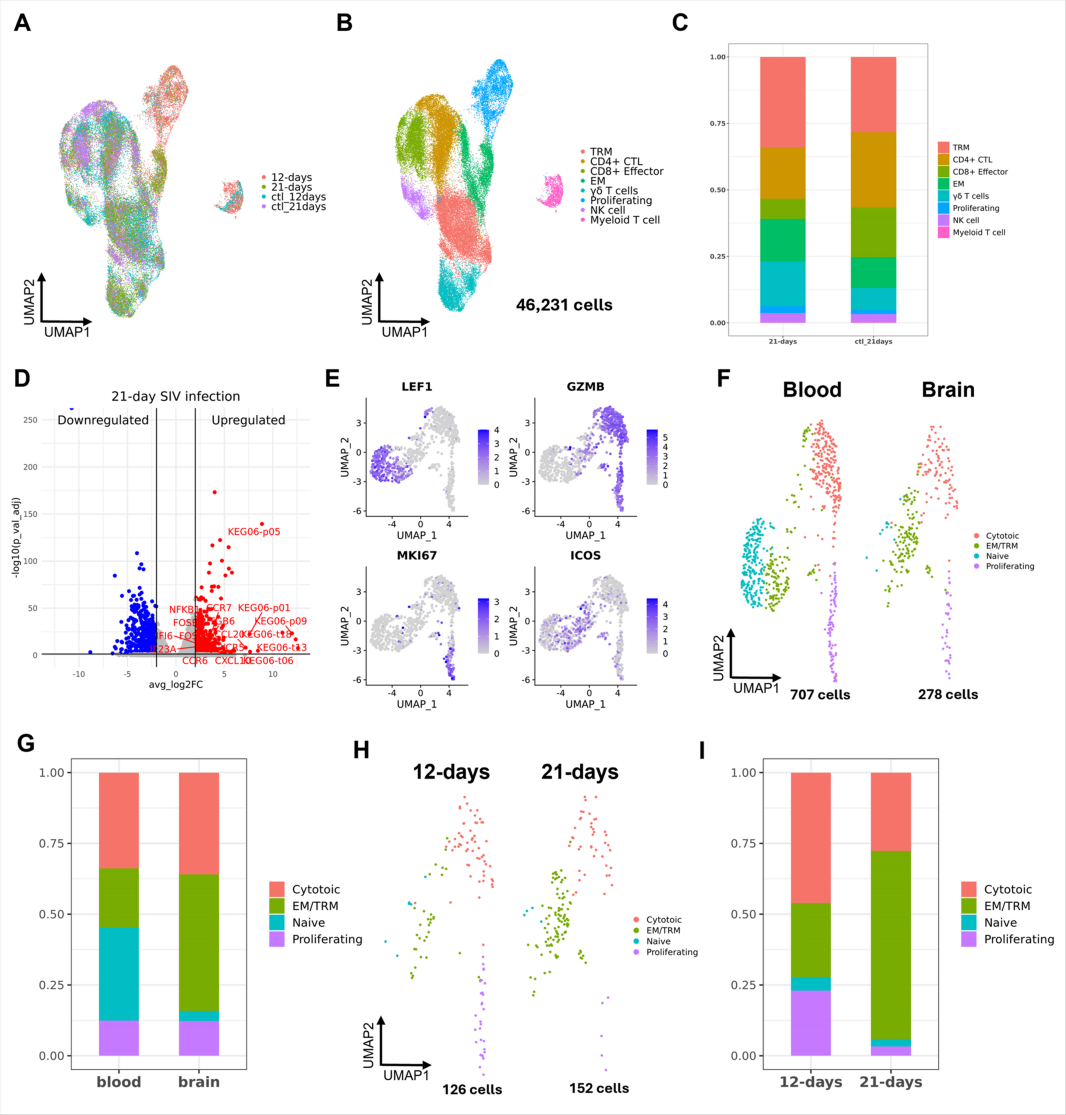
Brain lymphocyte comparison between 12 days and 21 days of acute SIV infection. **(A)** The UMAP projection of 46,231 T lymphocytes from two scRNA-seq datasets. The 12-day dataset included three infected animals (12 days) and three uninfected animals (ctl_12 days). The 21-day dataset included four infected animals (21 days) and two uninfected animals (ctl_21days). **(B)** The UMAP projection of 46,231 T lymphocytes was clustered into 8 distinctive lymphocyte clusters. **(C)** The relative contribution of each identified cluster in uninfected (ctl_21days) and 21-day acute-infected brain lymphocytes. **(D)** The genes were upregulated and downregulated for brain lymphocytes in 21-day infection compared to 12-day infection. The lymphocytes, excluding Myeloid T cells from 12-day and 21-day infections, were aggregated for a pseudobulk analysis. The statistical test for marker detection was the DEseq2 with parameters as adjusted p-value < 0.05 and absolute log_2_FC > 2. **(E)** Characterization of SIV+ T cells from 12-day infected brain and blood samples and 21-day infected brain samples. **(F)** The UMAP projection of 985 SIV+ T cells from 12-days and 21-days infected brains and 12-days infected bloods. **(G)** The relative contribution of each identified SIV+ T cell cluster in blood and brain samples. **(H)** The UMAP projection of 278 SIV+ T cells from 12-days and 21-days infected brains. **(I)** The relative contribution of each identified SIV+ T cell cluster in 12-day and 21-day brain samples.

To better understand the lymphocyte responses at different time points of acute SIV infection, we integrated a dataset (31) studying responses of brain lymphocytes in 21 days of acute SIV infection. The longer infection time in their study compared to ours (12 days) allowed for a comparison of lymphocyte responses at different points of acute SIV infection. The data integration of 46,231 lymphocytes from 12 animals (three for 12-day infection, four for 21-day infection, and five for uninfected controls) followed the same pipelines as our other analyses. The batch effect between samples was corrected, and the samples from the two datasets were combined (Fig. 8A). The clustering generated eight cell clusters. The clusters from the integrated dataset were characterized by mapping the annotations from the 12-day infection data and finding DEGs for each new cluster **(Suppl. Table. 10)**. Both methods confirmed a consistent annotation on this new integrated dataset (Fig. 8B).

We examined the lymphocyte population changes in 21-day infection. Except for the Myeloid-T cell cluster, the other T cell phenotypes found in 12-day infection were all found in 21-day infection. Consistent with our 12-day infection (Fig. 3J), the CD4 + CTLs decreased, effector memory (EM) T cells increased, and NK cells were unchanged in the 21-day infection (Fig. 8C). However, the decrease of CD8 + effector T cells and increased TRM and γδ T cells in the 21-day infection was the opposite of our 12-day infection. This could be caused by the variations between different datasets. Then, we determined the transcriptomic differences of all brain lymphocyte clusters between 12-days and 21-days SIV infection by performing DEG analysis at pseudobulk level (Fig. 8D). We found that the longer acute infection led to the upregulation of many mitochondrial genes, indicating increased cellular respiration and possibly response to stress. In addition, the lymphocytes in 21-day infection also upregulated several chemokine receptors (e.g. CXCR5, CCR6, and CCR7), cytokines (e.g. IL23A), and transcription factors related to immune activation (e.g. FOS, FOSB, and NFKB1) compared to 12-days infection, suggesting an enhanced activation state of those cells.

To validate our findings for infected cells, we reclustered the SIV + lymphocytes found in the blood (707 cells) and brain (126 cells) from the animals at 12 days p.i., and those found in the brain (152 cells) at 21 days p.i., totaling 985 cells. Because the 21-day infection dataset did not include Myeloid-T cells, the SIV + cells in this cluster from the 12-day infected animals were excluded from the analysis. The characterization showed naïve, cytotoxic, proliferating, and EM/TRM phenotypes. The cells in cytotoxic and proliferating T cell clusters were CD4 + CTLs. Interestingly, some cells in EM/TRM cluster also showed an upregulation of activation and cytotoxic genes (e.g. ICOS, CD28, and GZMK), indicating they might be under activation process (Fig. 8E **and Suppl. Table. 11**). The comparison of infected lymphocytes between blood and the brain still did not show the presence of CD4 + naïve T cells (Fig. 8F and 8G), consistent with what we found in the previous (Fig. 6E). This further highlighted that the longer infection time of acute SIV infection still primarily allowed for the infiltration of CD4 + CTLs compared to other CD4 + T cells. However, compared to the 12-day infection, there were more SIV + EM/TRM and fewer SIV + proliferating T cells in the brain of the 21-day infection **(Fig.s 8H and 8I)**, indicating a different pattern of infection in the brain lymphocyte populations.

## Discussion

The parenchyma of the CNS was long thought to be an immune-privileged site, shielded by tight cellular barriers from blood and cerebrospinal fluid (CSF), making it inaccessible to T cells. However, it is increasingly recognized that lymphocytes and particularly T cells phenotypes can be found in the brain parenchyma and perivascular space under physiological conditions. Most of these T cells are of the CD8 + phenotype (65, 67), in contrast to the predominance of CD4 + T cells found in CSF (68). The predominance of CD8 + T cells in the brain parenchyma and perivascular space are likely imperative in CNS immune surveillance, particularly to viral infection. In HIV infection, the CD8 + T cells also dominate the CSF, resulting in a lower CD4/CD8 T cell ratio (69). Infiltration of CD8 + T cells to the brain and CSF can contribute to the clearance of virus and infected brain cells. However, it might also trigger severe neurocognitive impairment and cerebral inflammation (28, 70). Although CD8 + T cells are the primary phenotype found in the brain under infection, the initiation of CNS infection is mostly caused by CD4 + T cells. Consistent with what has been reported, fewer CD4 + T cells were found in our brain lymphocyte dataset compared to CD8 + T cells, which include uninfected and 12-day SIV-infected rhesus macaque brain samples. Our scRNA-seq analysis found that those brain CD4 + T cells were primarily in two cell clusters: a tissue-resident memory (TRM) T cell cluster and a cytotoxic T cell cluster.

The TRM cells in the brain and other non-lymphoid tissues were generally considered as the cells that differentiate from circulating effector/effector memory T cells that encounter antigens during infections, vaccinations, or inflammatory events (71, 72). The integration analysis of brain and blood lymphocytes performed in this study also showed that some of the TRM cells in the brain (likely CD4+) shared transcriptomic profiles with CD4 + EM in the blood. (Fig. 7D) Given their memory, TRM cells can respond to antigens reencountering quickly (73, 74). The TRM cells in the brain have been found to contain both CD4 + and CD8 + T cells, and they share many similarities in their surface markers (54). The inseparable CD4 + TRM and CD8 + TRM T cells in the TRM cluster showed in our study **(Fig.s 3A and 3D)** indicated the transcriptomic convergence of those two brain TRM T cell phenotypes.

The TRM cells were dramatically depleted in 12-day SIV infection, inconsistent with a rapid increase of TRM (CD8+) reported in virus infection (75, 76). This might be because most of the depleted cells in our study were CD4+, and CD8 + TRM in infected brains changed their transcriptomic profile by upregulating proliferating genes, so those cells were clustered to proliferating T cell cluster instead of TRM cluster. Indeed, compared to uninfected controls, the proliferating T cell clusters had an over 10-fold increase in 12-day SIV infection, and most of the cells in the proliferating T cell cluster were CD8+. The analysis of 21-day SIV infection for brain lymphocytes showed restoration of the TRM population (slightly increasing) but decreasing proliferating T cells. Therefore, the brain TRM cells might be under quick proliferation to gain cytotoxicity and persist in the brain after exposure to SIV insults. The CD4 + TRM in the brain and CD4 + EM in the blood were susceptible to SIV infection, with a high percentage of SIV + cells (Fig. 6A and 6B). The high expression of LFA-1 in CD4 + EM also suggested their potential contributions in seeding the SIV/HIV to the CNS. Interestingly, the comparison of SIV + T cells in the brain with 12-day and 21-day SIV infection showed that the longer infection time in acute SIV infection dramatically promoted the SIV expansion in TRM, indicating they might serve as one of the brain reservoirs for HIV/SIV.

As another primary CD4 + lymphocyte population in the brain, CD4 + CTLs were found to decrease in both 12-day and 21-day acute SIV infection. This lymphocyte phenotype also could be found in the peripheral blood of infected and uninfected animals. Unlike its change in the brain, it was increased in the blood, which could be attributed to the absence of CD8 + T cells and NK cells in the analysis of blood samples. Since the CD4 + CTLs also had high expression of cytotoxic and activation genes, it is challenging to distinguish them from CD8 + CTLs. However, in this study, we found that, in general, CD4 + CTLs expressed low GZMA and GZMK genes, which were highly expressed in CD8 + CTLs. In another study, the transcriptomic profiling of CD4 + CTLs, CD8 + CTLs, and NK cells found that the transcripts differentiating CD8 + CTLs and CD4 + CTLs were primarily those shared between CD8 + CTL and NK cell (48). Our DEG analysis for CD4 + and CD8 + CTLs in the brain also showed that CD8 + CTLs highly expressed some NK cell markers (e.g. KLRC3, GNLY, and FCGR3), which could not be seen in CD4 + CTLs. The increased cytotoxicity in CD4 + CTLs compared to other CD4 + T cell phenotypes suggest its enhanced defending ability. Indeed, the CD4 + CTLs have been widely reported to contribute to virus clearance in numerous infectious diseases (77–82). Given of this, the concept of HIV-specific CD4 T cell response was proposed for controlling HIV replication in the absence of antiretroviral therapy(83, 84). However, given their longevity, the CD4 + CTLs were also found to serve as reservoirs for HIV-1(85). Our study here contributes to understanding the roles of CD4 + CTLs in the context of acute SIV brain infection. We found that CD4 + CTLs in both brain and peripheral blood were easier to get infected, which might be because they had higher CD4 and CCR5 expression than other CD4 + T cells (Fig. 6C and 6D). They also had enhanced chemotactic ability with high expression of VLA-4 and LFA-1 to seed the CNS infection. Therefore, the CD4 + CTLs might be the primary “Trojan horse” T cells that infect the brain in acute SIV/HIV infection.

A Myeloid-T cell cluster expressing both myeloid and lymphoid cell gene markers was identified in the brain of both acute infected and uninfected rhesus macaques, although they greatly increased in acute infection. To validate this observation is caused by real biological processes instead of technical artifacts, we established an MDM-T cells cocultivation system. Like what we observed the Myeloid-T cells in both infected and uninfected animals, the macrophage-T (Mac_T) cells could be observed on scRNA-seq in both apoptotic and non-apoptotic groups. Surprisingly, the cells in non-apoptotic groups had a larger proportion of this cluster, which is likely due to the accelerated Jurkat cell degradation during apoptosis. By comparing the cells in Mac_T cluster with a no-cocultivation control group, we found that the chemokines might serve as markers to distinguish the real Mac_T cells and technical doublets caused by scRNA-seq library preparations. Indeed, chemokines play an important role in recruiting other immune cells, activating macrophages and enhancing their phagocytic ability(85). Therefore, the upregulation of chemokines (e.g. CXCL3, CCL4, CCL3, CXCL2, CXCL1 etc.) might be the driving force for macrophages-T cell interactions. By applying this finding to the myeloid-T cell cluster in the brain of rhesus macaque, we found that over 50% of the cells in this cluster had co-expression of chemokines CCL3 and CCL4 and ~ 92% of cells had expression of at least one of those two chemokines. This suggests most cells in Myeloid-T cells are not technical artifacts but have biological meanings. Interestingly, the Myeloid-T cell cluster contained 22 SIV + cells, which represents 2% of the total cells in this cluster. Given the average infection rate in brain lymphocytes is ~ 1.4%, the high infection rate in Myeloid-T cluster suggests that the interactions between infected lymphocytes and brain myeloid cells efficiently amplify brain infection. It was found that all viruses could be effectively cell-to-cell transferred to macrophages from CD4 + T cells (86–89), and the cell-to-cell infection mechanism for HIV-1 transmission includes brain myeloid cells engulfing (89) or fusing (86, 87) with HIV-1-infected CD4 + T cells.

This study comprehensively showed the transcriptomic profile of brain lymphocytes responding to a 12-day acute SIV infection. By comparing with the blood CD4 + T cells, we identified the primary CD4 + T cell phenotypes that carry the SIV/HIV to the CNS. Further characterization and analysis showed that the chemotactic ability and expression of virus entry receptors (e.g., CD4 and CCR5) in the peripheral lymphocytes were highly correlated with their ability to seed CNS infection. Our scRNA-seq data showed that LEF-1 and VLA-4 were highly expressed in the lymphocyte phenotype in both blood and brain, indicating their essential roles in mediating the HIV/SIV invasion of the brain. However, more adhesion molecules and chemokine receptors might also be involved and worth further study to benefit therapeutic development. The integration of brain and blood lymphocyte datasets partially revealed the transcriptomic alternations of some lymphocytes (CD4+) once they enter the brain. The unique brain milieu could reshape those peripheral CD4 + T cells to adapt to the brain environment. However, because the CD8 + T cells were absent in the blood samples, the connection of CD8 + T cells in the brain and blood was lacking in this study. Additionally, we found that distinguishing between brain CD4 + and CD8 + T cells was challenging using scRNA-seq data. Many brain T cells in our dataset and the 21-day infection dataset (31) were neither CD4 + nor CD8+, which could also be attributed to the protein expression of those genes being more abundant than RNA expression (90). Therefore, the characterization of those brain lymphocytes might be further improved when the protein expression profile could be integrated with the RNA expression profile(85). Finally, given the advantage of large publicly available scRNA-seq data in the area of studying HIV/SIV infection, we also had a longitude comparison of brain T cell responses at different time points of acute SIV infection. Therefore, we believe the deposition of sequence data and metadata from our studies in publicly accessible databases can further benefit this society and enable the building of larger analyses with more subjects.

## Data Availability

The scRNA-seq data have been deposited in NCBI GEO accession number GSE293543. The code used for analyzing the data in this study was deposited in this GitHub repository: https://github.com/Howard-Fox-Lab/Brain_lymphocytes_acute_SIV_infection. All other data are in the Supplemental Tables or is available from the corresponding author upon request.
